# Bioactive Constituents and Pharmacological Properties of Lavender Essential Oil: Molecular Mechanisms, Current Evidence, and Future Perspectives

**DOI:** 10.3390/ijms27156931

**Published:** 2026-08-01

**Authors:** Ketong Chen, Yu Liu, Jinhang Nie, Guolong Wang, Junsong Xiao, Hua Wu

**Affiliations:** 1 School of Light Industry Science and Engineering, Beijing Technology and Business University, Beijing 100048, China; chenketong_06@163.com (K.C.); lyu7277@163.com (Y.L.); 15674815714@163.com (J.N.); 2 School of New Materials and Future Technologies, Beijing Technology and Business University, Beijing 100048, China; wangguolong_mortal@163.com; 3 School of Food and Health, Beijing Technology and Business University, Beijing 100048, China

**Keywords:** lavender essential oil, *Lavandula angustifolia*, linalool, linalyl acetate, antimicrobial activity, anti-inflammatory activity, antioxidant activity, neuropharmacology, wound healing, molecular mechanisms

## Abstract

Lavender essential oil (LEO) possesses broad therapeutic potential, yet translating empirical knowledge into evidence-based medicine is hindered by the descriptive literature that conflates isolated monoterpene mechanisms with whole-oil efficacy. Addressing these critical shortcomings, this review presents a target-centric synthesis that explicitly delineates the molecular pathways of pure constituents (e.g., linalool, linalyl acetate) versus intact LEO matrices and standardized oral preparations (Silexan™). We systematically integrate LEO’s multi-target signaling networks across neuroregulatory, analgesic, anti-inflammatory, and skin-regenerative domains, while critically examining how genetic taxonomy, environmental stress, and green extraction technologies dictate chemotype diversity. Furthermore, we candidly address current evidence limitations, including pre-clinical predominance, publication bias, and clinical trial narrowness. Finally, we outline actionable translational strategies centered on chemotype-driven standardization, smart delivery systems, and a dual pharmacological paradigm that valorizes non-volatile processing byproducts. This synthesis establishes a rigorous theoretical foundation for LEO’s clinical translation and pharmaceutical standardization.

## 1. Introduction

The genus *Lavandula* is an important group within the family Lamiaceae, comprising approximately 39 species and more than 400 registered cultivars [1]. Plants of this genus are generally characterized by erect inflorescences, green to gray-green leaves, and shrubby growth habits. Native to the Mediterranean region, *Lavandula* species are currently distributed extensively across southern Europe, North Africa, East Africa, southwestern Asia, western Iran, and parts of India. As traditional medicinal and aromatic plants, lavender-derived preparations have been widely used since the 19th century in aromatherapy and as complementary approaches for alleviating inflammation, infections, and emotion-related symptoms [2,3,4,5]. Lavender essential oil (LEO), a pale yellow volatile liquid with a characteristic herbaceous floral aroma, is primarily obtained from the aerial parts of lavender plants during the flowering stage through extraction methods such as steam distillation (SD) and hydrodistillation (HD) [6]. Due to its diverse biological activities and favorable application characteristics, LEO has been extensively used in pharmaceuticals, aromatherapy, perfumery, food flavoring, and cosmetics, and it has become one of the most widely utilized natural aromatic agents worldwide [7,8,9,10,11,12,13,14,15].

In recent years, several comprehensive reviews have documented the botanical origins, general phytochemical composition, and broad biological effects of LEO. For instance, classic and recent literature (e.g., Cavanagh & Wilkinson [2,5]; de Melo Alves Silva et al. [16]; Batiha et al. [17]; Jiang et al. [18]) has broadly surveyed the general pharmacology, cross-disciplinary applications, and clinical efficacy of lavender preparations. However, despite this wealth of general descriptive literature, a critical knowledge gap remains: Most existing reviews compile biological effects primarily as broad, activity-based lists (“X exhibits Y activity”) rather than systematically mapping out the precise molecular targets and underlying signaling networks. Furthermore, earlier works frequently blend findings from isolated single terpenes in non-lavender matrices with evidence from whole LEO, thereby obscuring the true mechanisms of the intact essential oil and overlooking the impact of chemotype variability.

To address these limitations, this review provides an updated, target-centric synthesis of LEO pharmacology. Rather than offering a simple descriptive compilation, we structure the mechanistic framework around specific molecular targets and interconnected signaling pathways: (1) anti-inflammatory and antioxidant axes involving NF-κB, MAPK, and Nrf2/HO-1 networks; (2) neuroregulatory effects mediated via GABA_A_, NMDA, and 5-HT_1A_ receptors; (3) nociceptive modulation targeting TRPA1 ion channels and the COX-2/catecholaminergic axis; and (4) cutaneous tissue regeneration mediated by ectopically expressed olfactory receptors (e.g., OR2AG2, OR10A6, OR51E2) and growth factor cascades. Crucially, this review explicitly distinguishes between evidence derived from whole LEO and that inferred from isolated constituents. In addition, we introduce new perspectives regarding a “dual pharmacological system” (valorizing non-volatile biomass residues alongside volatile oils) and chemotype-based quality standardization. By defining this molecular-targeted scope, this work aims to clarify the scientific basis of LEO, resolve mechanistic ambiguities, and provide a rigorous theoretical blueprint for its translational development in pharmaceutical, cosmetic, and therapeutic applications.

## 2. Major Factors Affecting the Composition and Safety of LEO

### 2.1. Overview of the Main Constituents of LEO

The major lavender species used for LEO production can be classified into four groups: *Lavandula angustifolia* (English lavender, formerly known as *L. vera* or *L. officinalis*), which exhibits strong cold tolerance and comprises numerous cultivars; *L. stoechas* (French or Spanish lavender), a late-flowering dwarf shrub characterized by gray-green leaves and a strong aroma; *L. latifolia*, a lavender species widely distributed in the Mediterranean region; and *L. × intermedia*, a hybrid derived from the crossbreeding of *L. latifolia* and *L. angustifolia* [5].

The chemical composition of LEO is commonly characterized and quantified using gas chromatography (GC) or gas chromatography–mass spectrometry (GC-MS) [19]. LEO mainly consists of monoterpenes, sesquiterpenes, and oxygenated derivatives, among which alcohols, esters, oxides, and ketones represent the predominant chemical classes. The major volatile monoterpenes identified in LEO include linalool, linalyl acetate, 1,8-cineole, β-ocimene, terpinen-4-ol, and camphor. The major sesquiterpene constituents include caryophyllene and nerolidol. In addition, some LEO samples contain perilla alcohol and other terpene derivatives [20].

### 2.2. Factors Affecting the Chemical Composition of LEO

The biological activities of LEO are closely associated with its major volatile constituents and their interactions. For a long time, some studies assumed that different lavender varieties might possess similar chemical compositions and biological activities due to their comparable traditional applications. However, extensive chemical analyses have demonstrated substantial variability in the composition of LEO. These variations are primarily associated with plant species and cultivars, while extraction methods, geographical origin, harvesting time, plant parts, and climatic conditions also contribute significantly to the observed differences. Accordingly, LEO samples from different sources exhibit considerable variations in both the profiles and relative abundance of their major volatile constituents (Table 1) [21].

As shown in Table 1, the major chemical constituents of LEO vary substantially depending on plant species, geographic origin, and extraction methods [4,15,17,20,21]. In general, *Lavandula angustifolia* essential oil is predominantly characterized by high levels of linalool (typically 20–45%) and linalyl acetate (25–47%), which contribute to its gentle aromatic profile and low cutaneous toxicity [11,19,25]. In contrast, essential oils from *L. latifolia* and *L. stoechas* contain significantly higher concentrations of 1,8-cineole (up to 40%) and camphor (10–30%) [4,19,22]. These compositional differences directly influence both the pharmacological activities and safety profiles of the oils. While higher levels of camphor and 1,8-cineole may enhance antibacterial or decongestant properties, they also increase the potential for skin irritation and neurological toxicity [19,22]. Therefore, therapeutic effects and safety thresholds cannot be generalized across all lavender oils, highlighting the necessity of chemotype-specific evaluation when referencing Table 1 for clinical or industrial application.

#### 2.2.1. Effects of Genetic Resources on the Composition of LEO

The major constituents of LEO exhibit considerable variation among different *Lavandula* species. Carrasco et al. compared the chemical compositions of essential oils from *L. angustifolia*, *L. latifolia*, and two cultivars of *L. × intermedia* (‘Grosso’ and ‘Super’), revealing distinct differences in their volatile profiles. Among these samples, the essential oil from *L. angustifolia* showed the highest linalool content (55.6%), whereas the two *L. × intermedia* cultivars were characterized by linalyl acetate as the predominant constituent, with contents of 25.8% and 25.7%, respectively. In contrast, the essential oil from *L. latifolia* contained the lowest level of linalyl acetate (0.1%) but exhibited relatively high levels of camphor (11.6%) and 1,8-cineole (29.7%) [19]. Similarly, Msaada et al. investigated the chemical compositions of essential oils obtained from the aerial parts of *L. dentata*, *L. stoechas*, and *L. multifida* cultivated in Tunisia using GC-MS. Significant interspecific differences were observed among these species. The essential oil from *L. stoechas* exhibited a higher proportion of linalyl acetate (50%) than those from *L. dentata* (28.65%) and *L. multifida* (7.30%), whereas linalool was more abundant in the essential oil from *L. dentata* (47.30%) and *L. multifida* (50.05%). In addition, the essential oil from *L. multifida* showed a relatively high camphene content (10.06%) [22].

Beyond interspecific differences, substantial variations in chemical composition have also been observed among different cultivars within the same *Lavandula* species. Kıvrak et al. analyzed the chemical profiles of essential oils obtained from six *L. angustifolia* cultivars and two *L. × intermedia* cultivars cultivated in Türkiye and found significant differences in the contents of major volatile constituents among cultivars [11]. Adaszynska and Dzieciol identified 42 and 39 compounds from the essential oil obtained from the *L. angustifolia* cultivars ‘Blue River’ and ‘Ellagance Purple’, respectively. The linalool content in the essential oil from ‘Ellagance Purple’ (22.51%) was approximately twice that detected in the essential oil from ‘Blue River’. In contrast, the essential oil from ‘Blue River’ contained higher levels of ester compounds, with linalyl acetate and lavandulyl acetate contents of 23.16% and 10.73%, respectively, which were significantly higher than those in ‘Ellagance Purple’ (16.39% and 4.97%, respectively) [25]. Similarly, Insawang et al. investigated the chemical compositions of essential oils obtained from five *L. stoechas* cultivars grown in Thailand. Camphor, 1,8-cineole, and fenchone were identified as the predominant volatile constituents, and their relative abundances varied considerably among cultivars. The camphor content showed the following trend: ‘Avonview’ (42.47%) > ‘Major’ (35.30%) > ‘Snowman’ (28.82%) > ×viridis ‘St. Brelade’ (7.13%) > ‘White lavender’ (3.68%). The corresponding fenchone contents were 35.42%, 37.18%, 23.73%, 23.24%, and 30.94%, respectively, while the relative content of 1,8-cineole ranged from 7.83 to 33.86% [4].

In summary, genetic resources—encompassing both interspecific taxonomy and intra-specific cultivar variations—represent the primary determinant of LEO chemical profiles, particularly regarding the ratio of linalool to linalyl acetate [11,19,22,25]. Generally, *L. angustifolia* and *L. × intermedia* are characterized by high proportions of monoterpene alcohols and esters (linalool and linalyl acetate), defining their premium aromatic and biological quality [19,25]. In contrast, species such as *L. latifolia* and *L. stoechas* accumulate higher levels of ketones and oxides, notably camphor, 1,8-cineole, and fenchone [4,19,22]. Recognizing these genetically driven metabolic signatures is fundamental for selecting targeted plant materials tailored to specific therapeutic or commercial requirements.

#### 2.2.2. Effects of Extraction Methods on the Chemical Composition of LEO

HD is one of the traditional methods used for essential oil extraction. With the development of extraction technologies, various approaches based on different principles have been established, including SD, supercritical carbon dioxide extraction (SCE), hexane extraction, turbine hydrodistillation (THD), ultrasound-assisted steam distillation (US-SD), and several microwave-assisted extraction techniques. These microwave-based methods include in situ microwave-generated hydrodistillation (ISMH), microwave steam distillation (MSD), microwave hydrodiffusion and gravity (MHG), and microwave steam diffusion (MSDf). In addition, other extraction strategies have been developed, such as solvent extraction (SE), NaCl-assisted hydrodistillation (NaCl-HD), enzyme-assisted hydrodistillation (Enzyme-HD), micelle-assisted hydrodistillation (Micelle-HD), ultrasound-assisted hydrodistillation (US-HD), subcritical water hydrodistillation (SW-HD), and solvent-free microwave extraction (SFME) [26,27].

Studies have demonstrated that even when identical plant materials are used, the chemical composition and relative abundance of major constituents in LEO can vary substantially depending on the extraction method. Danh et al. compared the chemical profiles of essential oil obtained from *L. angustifolia* using HD, SCE, and hexane extraction. The results showed significant differences in the relative content of the major constituent linalool, with values of 53%, 43%, and 33% in the HD extract, SCE extract, and hexane extract, respectively. The content of another major compound, linalyl acetate, was comparable between the hexane extract (25.7%) and SCE extract (23.4%), whereas it accounted for only 9.3% in the HD extract. Compared with other extraction approaches, SCE produced an essential oil with an aroma profile closer to that of the original plant material, reduced thermal degradation, and enhanced antioxidant and antimicrobial activities [10].

Although SCE offers advantages such as high extraction efficiency, low impurity levels, rapid processing, and environmental compatibility, its relatively high equipment cost remains a major limitation for large-scale applications. Different extraction technologies can also alter the relative proportions of characteristic constituents in essential oils. For example, the essential oil obtained by Micelle-HD showed a relatively higher linalool content, whereas the essential oil obtained by SW-HD exhibited a higher α-terpineol content [28]. However, solvent extraction may introduce additional non-volatile or co-extracted compounds, such as coumarin and 7-methoxycoumarin, which are generally absent in essential oils obtained by HD [29].

Périno-Issartier et al. investigated the extraction of essential oil from *L. × intermedia* using eight different approaches, including HD, SD, THD, US-SD, MSD, SFME, MHG, and MSDf. Gas chromatography–mass spectrometry analysis revealed that linalool and linalyl acetate, as the major characteristic constituents, exhibited substantial variations in relative abundance depending on the extraction method. The linalool content ranged from 25.16% to 47.51%, whereas the linalyl acetate content ranged from 11.75% to 25.50%. Among the evaluated methods, the essential oil obtained by MHG showed the highest linalool content (47.51%), followed by those obtained by THD (46.70%), SFME (45.37%), and SD (38.79%). Notably, the essential oil obtained by MHG exhibited relatively high levels of both linalool (47.51%) and linalyl acetate (25.50%) [30]. Similarly, Kiran Babu et al. reported that during the extraction of essential oil from *L. angustifolia*, the highest linalyl acetate content was obtained using SCE (51.8%), followed by solvent extraction combined with HD (31.4%) and SD (26.8%) [29].

In addition to extraction techniques, operational parameters during the extraction process, including temperature, heating duration, enzymatic treatment conditions, and electrolyte supplementation, can also influence the chemical composition and extraction efficiency of essential oils. For example, NaCl-assisted and enzyme-assisted extraction approaches have been shown to improve extraction performance. As an electrolyte, sodium chloride may alter the properties of the aqueous phase and affect the partition behavior between volatile constituents and the water phase, thereby facilitating the release of volatile compounds. In contrast, cellulase can promote the liberation of intracellular volatile constituents by degrading plant cell wall structures. Based on these mechanisms, NaCl-HD and Enzyme-HD can enhance the extraction efficiency of essential oil to some extent [28,31].

Notably, the essential oil obtained by MSD and SFME contained slightly higher levels of linalyl acetate than linalool, whereas the opposite trend was observed for the essential oil obtained using other extraction methods. This phenomenon may be associated with the hydrolysis or thermal degradation of linalyl acetate during the extraction process, which can generate linalool and acetic acid. Conventional SD generally requires prolonged heating at relatively high temperatures, which may promote ester degradation and consequently increase the relative proportion of linalool in the resulting essential oil. In contrast, MSD and SFME involve shorter processing times, potentially reducing ester degradation and facilitating the preservation of linalyl acetate [28].

Extraction time is also an important factor influencing the chemical composition and extraction efficiency of LEO. Zheljazkov et al. evaluated the effects of hydrodistillation duration on the yield and chemical composition of essential oils obtained from dried lavender flowers. The results showed that the relative contents of 1,8-cineole and fenchone reached relatively high levels after 15 min of hydrodistillation, ranging from 6.4% to 35% and from 1.7% to 2.9%, respectively, and they subsequently decreased with prolonged distillation times. The relative content of camphor reached its highest level between 7.5 and 15 min (6.6–9.2%), whereas linalyl acetate exhibited the highest relative content after 30 min of distillation (15–38%). When the distillation time was extended to 60 min, the yield of LEO appeared to reach a plateau, with no further significant increase observed [32]. These findings indicate that extraction time is a critical parameter affecting both the chemical profile and yield of LEO. Therefore, appropriate optimization of distillation duration is necessary to achieve desirable essential oil quality during the preparation process.

Collectively, the choice and optimization of extraction technologies fundamentally dictate the thermolabile ester recovery and overall chemical fidelity of LEO [10,28,30,32]. Conventional steam distillation and hydrodistillation often promote the thermal degradation or hydrolysis of linalyl acetate into linalool and acetic acid due to prolonged thermal exposure [28]. Conversely, modern green extraction techniques, such as supercritical carbon dioxide extraction (SCE) and microwave-assisted hydrodistillation methods (e.g., MHG and SFME), significantly shorten processing duration and lower operating temperatures, thereby better preserving native ester profiles and reducing artifact formation [10,28,30]. Consequently, standardizing extraction parameters is imperative to ensure batch-to-batch chemical reproducibility.

#### 2.2.3. Effects of Cultivation Environment and Management Practices on the Chemical Composition of LEO

Environmental factors, including altitude, climate, soil properties, and water availability, can influence the yield and chemical composition of LEO. Woo et al. compared 33 essential oil samples obtained from *L. angustifolia* cultivated in different countries and found significant geographical variations in the relative ratio of linalool to linalyl acetate. The ratio followed the following trend: Australia > France > Korea [33]. Bajalan and Pirbalouti analyzed the chemical compositions of essential oil obtained from 10 different geographical locations of *L. × intermedia* in the Zagros region of western Iran using gas chromatography–mass spectrometry. The results demonstrated considerable differences in the proportions of major constituents among samples from different locations. The content ranges of 1,8-cineole, borneol, and camphor were 31.64–47.94%, 17.11–26.14%, and 8.41–14.40%, respectively [34]. Furthermore, Miomir et al. investigated commercial essential oil samples obtained from *L. angustifolia* originating from Budva, Rovinj, and Montenegro, and they identified 100 chemical constituents in total. Although all samples were commercially available products, considerable differences in their major chemical profiles were observed. The Budva sample was mainly characterized by linalool (27.32%), borneol (20.24%), and β-phellandrene (19.10%), whereas the Rovinj sample exhibited a higher linalool content (47.67%) and contained 11.82% camphor. In contrast, the Montenegro sample was primarily composed of camphor (21.23%) and linalool (19.92%) [23]. Collectively, these studies indicate that environmental conditions associated with cultivation regions may contribute to variations in the chemical composition of LEO.

In addition to long-term geographical differences, annual climatic fluctuations may also affect the chemical composition of essential oils. Détár et al. investigated the characteristics of essential oil obtained from six *L. angustifolia* cultivars and two *L. × intermedia* cultivars harvested during the summers of 2017 and 2018 in Hungary. The results showed that, in 2018, a year with higher precipitation, the relative content of linalool decreased, whereas the relative content of linalyl acetate increased across different cultivars [35]. These findings further demonstrate that both long-term cultivation environments and annual climatic variations may influence the chemical composition of LEO.

The chemical composition of essential oils may also vary among different plant parts. Talal et al. analyzed the chemical profiles of essential oil obtained from the flowering tops, leaves, and entire aerial parts of *L. coronopifolia* using gas chromatography–mass spectrometry. The results demonstrated distinct distribution patterns of major constituents among different plant tissues. The relative content of linalool exceeded 40% in both flowering top and leaf essential oils, whereas the essential oil obtained from the entire aerial part exhibited a relatively high 1,8-cineole content (approximately 25.4%), which was about three times higher than that detected in the leaf and flowering top samples [24].

Harvesting time is another important factor affecting the chemical composition of LEO. Yalcin et al. reported that both harvesting time and plant tissues influenced the relative abundance of major constituents, including linalool, camphor, 1,8-cineole, terpinen-4-ol, and isoborneol, in essential oil obtained from *L. officinalis*. Linalool was detected in all examined tissues, with the highest content observed in stems harvested at noon (27.88%), followed by stems collected in the morning (25.23%) and evening (24.73%). In flowers, linalool contents were the highest in samples collected in the evening (26.09%), followed by those collected at noon (18.38%) and in the morning (17.59%). The linalool content in leaves was comparatively lower (1.32–5.09%), with the highest level detected in morning-harvested samples. The distribution of other major constituents also varied among plant tissues. The highest 1,8-cineole content was detected in leaves (12.32%), whereas stems showed the lowest level (0.59%). Camphor contents in leaves (0.71–2.58%) were lower than that in stems and flowers, with the highest camphor concentration observed in flowers harvested at noon (18.86%), followed by stems harvested at noon (3.96%). Terpinen-4-ol was mainly detected in stems and flowers but was absent in leaves. In addition, isoborneol exhibited the highest content in leaves collected at noon (19.27%) [36].

The chemical composition of essential oil may also vary with plant developmental stages, as the distribution of secondary metabolites within the same plant organ can change throughout different growth periods. Branislav et al. reported that the essential oil obtained from immature *L. angustifolia* leaves collected in April in Belgrade was mainly characterized by β-phellandrene. By May, the relative content of β-phellandrene decreased, whereas the concentration of 1,8-cineole increased and remained relatively high in mature leaves, particularly in overwintered leaves [37].

Collectively, the chemical composition of LEO is influenced by multiple factors, including cultivation region (such as altitude, climate, soil properties, and water availability), plant parts, harvesting time, and developmental stage. These factors collectively contribute to variations in essential oil quality characteristics and chemical profiles and, therefore, should be carefully considered during raw material selection and quality control [23,24,33,34,35,36,37]. Mechanistically, these environmental stress dynamics and developmental shifts govern terpene biosynthesis by modulating key synthases [35,37]. To overcome these multifactorial variations and ensure reproducible therapeutic efficacy, future sourcing must transition from geographical labeling toward strict, chemotype-based standardization [33,34].

### 2.3. Safety of LEO

Due to its extensive biological activities, LEO has attracted considerable attention from international regulatory agencies and relevant organizations. The U.S. Food and Drug Administration (FDA) has classified lavender oil as a substance generally recognized as safe (GRAS) under the Code of Federal Regulations (21 CFR 182.20), allowing its use as a natural flavoring ingredient in food products. In addition, information from organizations, including the World Health Organization (WHO), the European Medicines Agency (EMA), and the European Scientific Cooperative on Phytotherapy (ESCOP), has highlighted the traditional applications of lavender preparations and their potential value in the complementary management of emotional symptoms such as anxiety and nervous tension [38].

Although current regulatory evaluations and scientific evidence generally support the safety of LEO when used appropriately, clinical studies and practical applications have indicated that it may still cause certain cutaneous adverse reactions. These reactions mainly include irritant contact dermatitis (ICD) and allergic contact dermatitis (ACD). ICD is generally associated with local irritation or cytotoxic effects caused by exposure to high concentrations of essential oil, whereas ACD is primarily related to immune sensitization induced by oxidation products formed during essential oil storage and exposure [39].

Regarding cytotoxic effects, Prashar et al. reported that essential oil obtained from *L. angustifolia* exhibited cytotoxicity toward human skin cells at concentrations above 0.25%. Its major constituents, linalyl acetate (51%) and linalool (35%), were suggested to contribute to membrane damage, with linalyl acetate showing a stronger cytotoxic effect than linalool [40]. Such reactions are typically concentration-dependent and may manifest as local stinging, burning sensations, and erythema, which are consistent with the clinical features of irritant contact dermatitis.

Regarding allergic contact dermatitis (ACD), Bingham et al. retrospectively analyzed patch test data collected by the Skin and Cancer Foundation of Victoria, Australia, from 1993 to 2017. During the study period, 2178 patients underwent patch testing for lavender-related allergens. Among them, 49 patients exhibited 58 positive reactions, corresponding to an overall positivity rate of 2.2%, and 27 patients were diagnosed with ACD. The most common sources of lavender exposure were personal care products and essential oils. Among patients with ACD, 74% underwent patch testing with lavender absolute, of whom 90% showed positive reactions. The study indicated that lavender-associated ACD is relatively uncommon; however, due to potential exposure through personal care products and essential oils, its skin sensitization potential should still be considered [41].

Notably, storage conditions may influence the sensitization potential of essential oils. Prolonged exposure to air can induce autoxidation of major LEO constituents, particularly linalool and linalyl acetate, resulting in the formation of oxidation products with strong sensitizing properties and consequently increasing the risk of skin sensitization [41,42,43,44,45]. An international multicenter study using a 6.0% oxidized linalool preparation in petrolatum demonstrated that 6.9% of 2900 participants showed positive patch test reactions, suggesting that prolonged or repeated exposure to low concentrations of oxidized linalool may increase the risk of ACD development [42,43]. In addition, linalyl acetate, which shares structural similarities with linalool, can also undergo oxidation in air to generate potentially sensitizing oxidation products [46]. Therefore, excessive concentration, prolonged exposure, frequent topical application, and improper storage conditions may collectively increase the likelihood of LEO-associated cutaneous adverse reactions.

Considering the potential safety concerns described above, international quality management of LEO primarily focuses on the standardization of chemical composition and product quality control. Linalool and linalyl acetate, as the major volatile constituents of LEO, are not only closely associated with its characteristic aroma profile but are also widely used for evaluating product consistency and quality as important quality markers [47].

To further regulate the quality requirements and international trade of LEO products, the International Organization for Standardization (ISO) established the ISO 3515:2002 standard in 2002 [48]. This standard specifies the characteristic chemical composition ranges of LEO derived from natural wild lavender populations (French populations) and clonal cultivars of *L. angustifolia*. The corresponding quality criteria are summarized in Table 2. Currently, the quality evaluation of LEO mainly relies on the relative proportions of characteristic constituents, including camphor, linalool, linalyl acetate, and lavandulyl acetate.

Further studies integrating comprehensive chemical profiling, toxicological assessments, and clinical investigations are required to better elucidate the variations in safety and biological activity among LEOs derived from different lavender species and genetic resources. Commercial production and application of LEO should comply with ISO standards and other relevant quality regulations to improve product consistency and facilitate its safe utilization in the food, pharmaceutical, and cosmetic industries.

## 3. Biological Activities of LEO

**Figure 1:**
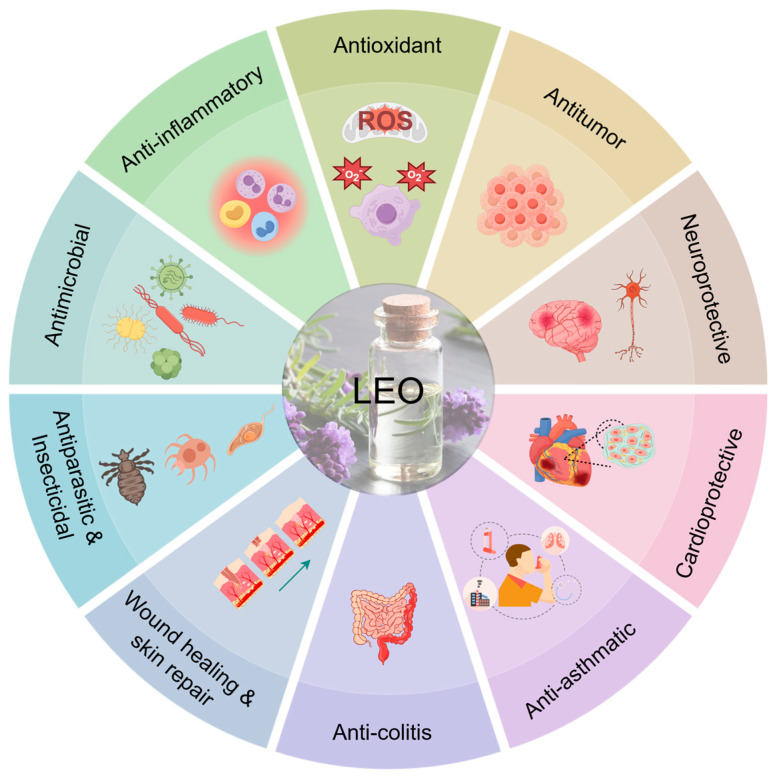
Biological activities and potential therapeutic effects of lavender essential oil (LEO). This schematic illustration summarizes the reported biological activities of LEO, including antioxidant, anti-inflammatory, antimicrobial, antiparasitic and insecticidal, wound healing and skin repair, anti-colitis, anti-asthmatic, cardioprotective, neuroprotective, and antitumor effects. The central image represents LEO, while the surrounding sectors indicate its diverse pharmacological activities and associated biological targets or processes.

LEO exhibits a wide range of biological activities (Figure 1). These effects are likely associated with its complex chemical composition and the regulatory actions of its major volatile constituents on multiple molecular targets and signaling pathways. This section summarizes the potential mechanisms underlying the diverse biological effects of LEO from the perspectives of molecular targets and signaling networks, with a particular focus on its major bioactive constituents, including linalool, linalyl acetate, and 1,8-cineole. It is worth noting that while some mechanistic insights are derived directly from intact lavender essential oils, others are based on isolated main constituents (e.g., linalool, linalyl acetate, terpinen-4-ol) or studies in non-lavender matrices. In the following sections, evidence from whole LEO is explicitly distinguished from inferences drawn from isolated single compounds.

### 3.1. Antibacterial Activity and Applications of LEO

#### 3.1.1. Antibacterial Mechanisms and Potential Modes of Action of LEO

**Figure 2:**
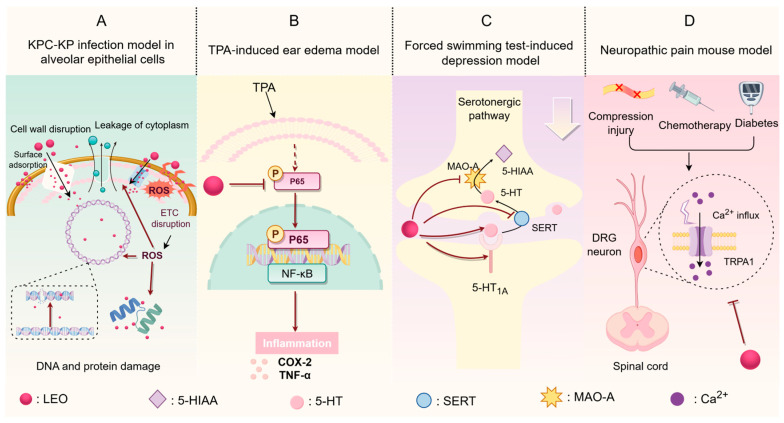
Overview of the biological activities and underlying mechanisms of lavender essential oil (LEO). (**A**) Antibacterial mechanisms of LEO against bacterial pathogens, including adsorption to the bacterial surface, cell wall disruption, electron transport chain (ETC) disruption, ROS generation, and oxidative damage to DNA and proteins; (**B**) anti-inflammatory mechanisms of LEO involving inhibition of NF-κB signaling activation and subsequent reduction in inflammatory responses, including the regulation of inflammatory mediators such as COX-2 and TNF-α; (**C**): antidepressant mechanisms of LEO associated with modulation of serotonergic neurotransmission, including regulation of serotonin (5-HT) metabolism, inhibition of serotonin degradation by monoamine oxidase A (MAO-A), and modulation of serotonin transporter (SERT) and 5-HT_1A_ receptor signaling; (**D**) analgesic mechanisms of LEO involving modulation of pain-related pathways, including regulation of transient receptor potential ankyrin 1 (TRPA1)-mediated cation influx in neuropathic pain models.

LEO exhibits antibacterial activity against a broad range of Gram-positive (G^+^) and Gram-negative (G^−^) bacteria. Based on differences in cell wall and cell membrane structures, bacteria are generally classified into Gram-positive bacteria (G^+^) and Gram-negative bacteria (G^−^). G^−^ bacteria possess an outer membrane containing lipopolysaccharides (LPSs), which may restrict the penetration of certain hydrophobic antibacterial compounds. However, numerous in vitro studies have demonstrated that LEO can inhibit the growth of both bacterial groups, although the reported minimum inhibitory concentration (MIC) and minimum bactericidal concentration (MBC) values vary depending on bacterial species, LEO chemical composition, and experimental conditions (Table 3). The antibacterial effects of LEO are unlikely to rely on a single molecular target; rather, they stem from multi-target engagement driven by the synergistic interactions of its major monoterpene constituents (such as linalool, 1,8-cineole, and camphor) within the whole essential oil matrix [49]. Recent evidence confirms that intact LEO directly induces membrane damage and oxidative stress in resistant bacterial strains [49]. The potential antibacterial modes of action can be categorized into three major aspects (Figure 2A):

**Disruption of Bacterial Membrane Integrity and Alteration of Permeability:** As highly lipophilic molecules, the combined volatile terpenes in whole LEO (as well as individual isolated constituents) interact with bacterial membrane lipids and partition into the phospholipid bilayer, compromising membrane stability and integrity. This physical perturbation increases membrane permeability, leading to the leakage of critical intracellular ions (e.g., potassium) and essential cellular constituents, which ultimately disrupts bacterial viability [58,59,60,61].

**Induction of Oxidative Stress:** The interaction of LEO constituents with bacterial membranes further interferes with cellular energy metabolism and electron transport networks. Direct experimental evidence demonstrates that whole LEO exposure triggers intracellular accumulation of reactive oxygen species (ROS) and impairs bacterial antioxidant defense enzymes (such as superoxide dismutase). Persistent oxidative stress subsequently causes irreversible damage to bacterial DNA, functional proteins, and metabolic machinery [49,62,63].

**Regulation of Quorum Sensing and Inhibition of Biofilm Formation:** Beyond direct physical and oxidative bactericidal action, both whole LEO and its constituent monoterpenes can interfere with quorum-sensing-associated signaling pathways, reduce cell surface adhesion capacity, and inhibit microbial biofilm maturation. These regulatory actions attenuate bacterial colonization and pathogenicity [64,65,66,67].

Collectively, LEO may exert antibacterial effects through multiple mechanisms, including disruption of bacterial membrane structures, induction of oxidative stress, and interference with quorum sensing and biofilm development. This multi-target mode of action highlights the potential value of LEO as a natural antibacterial agent. However, further systematic investigations are required to clarify its precise molecular mechanisms and to determine whether LEO can effectively contribute to reducing the risk of bacterial resistance.

#### 3.1.2. In Vitro Antibacterial Activity of LEO and Its Synergistic Effects with Antibiotics

Differences in bacterial cell envelope structures may influence antibacterial susceptibility to essential oil constituents. Specifically, the MIC values against the G^+^ bacteria *Arthrobacter bohemicus* and *Bacillus cereus* were 0.47% and 0.94%, respectively. In contrast, the MIC values against the G^−^ bacteria *Escherichia coli* and *Pseudomonas fluorescens* were 1.87% and 3.75%, respectively, while that against *Kocuria marina* was 1.87% [53].

It should be noted that antibacterial activity data reported in different studies vary considerably in terms of units of expression, including % *v*/*v*, mg/mL, and μL/mL, mainly due to differences in experimental designs and reporting approaches. Nevertheless, these findings collectively indicate the antibacterial potential of LEO. For more accurate comparisons and evaluations in future studies, standardized antibacterial testing protocols are required, including harmonization of bacterial inoculum concentrations, activity evaluation parameters, unit expression, and experimental conditions.

With the increasing challenge of antibiotic resistance, the combination of LEO with conventional antibiotics has attracted attention due to its potential synergistic effects. For example, essential oil derived from the Moroccan endemic lavender species *L. maroccana* exhibited inhibitory activity against several pathogenic bacteria, including *Staphylococcus aureus*, *Micrococcus luteus* (ATCC 10240), *Bacillus subtilis* (ATCC 9524), *Escherichia coli* (ATCC 8739), clinical isolates of *Klebsiella pneumoniae*, and *Pseudomonas aeruginosa* (DSM 50090). Among these strains, the inhibitory effects against *M. luteus* and *B. subtilis* were more pronounced, with MIC values of 2.302 mg/mL [52]. Furthermore, combination studies demonstrated that *L. angustifolia* EO combined with gentamicin enhanced antibacterial activity against *S. aureus* (ATCC 25923) and methicillin-resistant *S. aureus* (MRSA) [68]. These findings suggest that LEO may serve as a potential complementary strategy in combination with existing antibiotics to improve antibacterial efficacy.

#### 3.1.3. Formulation Optimization and Potential Applications of LEO

Although LEO exhibits antibacterial activity, its high volatility and relatively limited stability may restrict its further application. Therefore, formulation optimization has become an important strategy to improve its practical value. Microencapsulation technology can enhance the stability of essential oils and improve their antibacterial activity and duration of action. Studies have demonstrated that microencapsulated LEO exhibits stronger antibacterial activity against *E. coli*, *S. aureus*, and *B. cereus* compared with untreated essential oil [55]. Furthermore, Yuan et al. reported that encapsulation of LEO with hydroxypropyl-β-cyclodextrin reduced the MIC value to approximately one-third of that of free essential oil [56]. These formulation optimization strategies provide potential approaches for expanding the application of LEO in infection-related fields.

**Digestive and Respiratory Tract Infections:** In digestive system infections, diarrhea and foodborne diseases caused by enterohemorrhagic *Escherichia coli* O157:H7, *S. aureus*, *Listeria monocytogenes*, and antibiotic-resistant strains remain important public health concerns. Dadalioglu and Evrendilek demonstrated that *L. stoechas* EO cultivated in Spain exhibited inhibitory activity against these foodborne pathogens, with antibacterial activity ranked as follows: *S. typhimurium* > *S. aureus* > *L. monocytogenes* > *E. coli* O157:H7 [69]. Regarding respiratory tract infections, *L. angustifolia* EO has been reported to inhibit several respiratory pathogens, including *Streptococcus pyogenes* (*S. pyogenes*), *Streptococcus agalactiae* (*S. agalactiae*), *Streptococcus pneumoniae* (*S. pneumoniae*), *K. pneumoniae*, *Haemophilus influenzae* (*H. influenzae*), *S. aureus*, and *Stenotrophomonas maltophilia* (*S. maltophilia*). No obvious cytotoxic effects were observed under the experimental conditions [70].

**Oral Health and Local Microbial Ecosystems:** In oral health, periodontitis is a localized inflammatory disease affecting periodontal supporting tissues. Without timely intervention, it may result in connective tissue destruction and alveolar bone resorption, eventually leading to tooth loss. The development of periodontitis is closely associated with bacterial colonization and the formation of dental and gingival biofilms. Gursoy et al. reported that several plant essential oils, including *L. stoechas* EO, exhibited inhibitory effects against periodontal pathogens [57]. Recent studies have suggested that the major constituents of LEO, linalool and linalyl acetate, may act synergistically to influence microbial biofilm integrity, reduce biofilm adhesion, and regulate inflammation-related signaling pathways within mucosal microenvironments [71]. These mechanisms suggest that LEO may have potential applications in local infection-associated conditions, including denture stomatitis, mucositis, and postoperative wound care.

**Skin Wounds and Tissue Repair:** Wound healing is influenced by immune status and microbial infection. Bacterial colonization can aggravate local inflammatory responses and delay tissue repair, particularly in individuals with impaired immune function, such as patients with diabetes or obesity. Sienkiewicz et al. reported that LEO obtained from Poland effectively inhibited wound-associated pathogens, including *S. aureus*, *E. faecalis*, *E. coli*, *K. pneumoniae*, *E. cloacae*, and *A. baumannii* [50]. In addition to directly inhibiting pathogen growth, volatile constituents of LEO may contribute to local tissue repair by modulating inflammatory responses and supporting the wound healing process. Therefore, LEO may represent a potential candidate for applications in infection-associated skin injuries and wound care.

Collectively, LEO demonstrates diverse potential applications in bacterial infection control through its antibacterial activity, synergistic effects with antibiotics, and improved stability and sustained release properties achieved through formulation optimization. However, current evidence is primarily derived from in vitro studies and preliminary application investigations. Further studies using standardized evaluation systems and clinical investigations are required to validate its safety and efficacy, thereby providing stronger scientific support for the development of LEO-based functional formulations and supportive therapeutic applications.

### 3.2. Antifungal Activity and Applications of LEO

#### 3.2.1. Antifungal Mechanisms and Potential Interventions of LEO

Unlike bacteria, pathogenic fungi are eukaryotic organisms with complex and highly adaptable cell wall structures, which play essential roles in maintaining cellular morphology, structural integrity, and environmental adaptation. Therefore, the prevention and control of fungal infections generally present greater challenges compared with bacterial infections [72]. Despite these challenges, LEO and its major lipophilic monoterpenes exhibit notable antifungal activity. The MIC and minimum fungicidal concentration (MFC) values reported in different studies vary considerably depending on fungal species, LEO composition, and experimental conditions (Table 4). Current evidence suggests that the antifungal effects of LEO are mainly mediated through the following two mechanisms:

**Disruption of Fungal Cell Membranes and Inhibition of Morphological Transition:** The small volatile molecules present in LEO can interact with fungal membrane lipid structures, affecting membrane integrity and permeability and thereby causing intracellular leakage and disrupting essential cellular functions [16,77]. In addition, LEO and its bioactive constituents may inhibit the morphological transition of certain pathogenic fungi from yeast-like forms to filamentous forms, including the suppression of germ tube formation and hyphal growth, thereby reducing fungal invasive capacity [17,76].

**Inhibition of Spore Germination and Mycotoxin Production:** The bioactive components of LEO can inhibit fungal spore germination and interfere with oxidative metabolism and energy production pathways [6,17]. Furthermore, previous studies suggest that LEO may reduce the biosynthesis of certain mycotoxins by modulating fungal secondary metabolic pathways [78].

#### 3.2.2. In Vitro Inhibitory Activity of LEO Against Clinical Pathogenic Fungi

*Candida albicans* is a common opportunistic yeast associated with superficial mucosal infections and invasive fungal infections in hospitalized patients, and it is also one of the major pathogens responsible for vulvovaginal candidiasis [79]. D’Auria et al. reported that *L. angustifolia* EO exhibited inhibitory activity against all 50 clinical *C. albicans* isolates obtained from oropharyngeal and vaginal sources, with an MIC of approximately 1%. In comparison, linalool, one of its major constituents, showed stronger antifungal activity, with an MIC of approximately 0.3% [73]. Another comparative study demonstrated that *L. angustifolia* EO exhibited comparable or superior inhibitory effects against *C. albicans* compared with clotrimazole, and the difference between the two treatments became more pronounced with prolonged exposure time [80]. Furthermore, *L. vera* EO showed inhibitory activity against several clinically relevant *Candida* species, including *C. albicans*, *C. glabrata*, and *C. tropicalis*. Further investigations suggested that linalool may represent one of the major active constituents contributing to its antifungal effects [74]. In combination therapy studies, LEO obtained from two *L. angustifolia* cultivars (“Blue River” and “Elegance”) enhanced the inhibitory effect of fluconazole against *C. albicans* ATCC 10231, with the “Blue River” cultivar exhibiting a more pronounced synergistic effect [81].

Dermatophyte infections represent a common type of superficial fungal infection and remain a persistent public health concern. Among dermatophytes, *Trichophyton mentagrophytes* and *Trichophyton rubrum* are considered major causative pathogens. A study showed that LEO obtained from Farm Tomita, containing 38% linalool and 40% linalyl acetate, exhibited moderate inhibitory activity against these two dermatophyte species. Moreover, linalool alone showed an MIC of approximately 400 μg/mL [75].

#### 3.2.3. Applications of LEO in Environmental Fungal Control and Food Preservation

Due to its antifungal activity and recognition as generally recognized as safe (GRAS) by the U.S. Food and Drug Administration (FDA), LEO has shown potential applications in environmental microbial control and food preservation.

**Control of Environmental and Toxigenic Fungi:** *Aspergillus* and *Penicillium* species are among the most common fungal contaminants in food storage environments and indoor environments. Some species can produce mycotoxins, posing potential risks to food safety and human and animal health. Studies have demonstrated that *L. angustifolia* LEO can inhibit mycelial growth, conidial germination, and mycotoxin production of *Penicillium digitatum* [76]. Quantitative analyses have shown that LEOs from different sources generally exhibit stronger inhibitory effects against Aspergillus species than against Penicillium species [82,83]. In addition, LEO from Australia has been reported to inhibit the growth of airborne fungi, including Coprinellus and Ulocladium, suggesting its potential application in regulating indoor airborne microbial populations [84].

**Natural Preservation and Fruit and Vegetable Storage:** In the field of natural preservation, LEO exhibits inhibitory activity against various food-spoilage-associated yeasts, including *Torulaspora delbrueckii*, *Dekkera anomala*, *Pichia membranifaciens*, *Yarrowia lipolytica*, and *Zygosaccharomyces bailii*. Although its inhibitory activity is lower than that of lemon balm essential oil, its broad-spectrum effects against spoilage microorganisms indicate its potential as a natural preservative ingredient [85]. In addition, *L. officinalis* EO treatment has been shown to inhibit the growth of *Penicillium solitum* and contribute to the preservation of cheese quality during storage [86]. Regarding fruit and vegetable preservation, LEO treatment can reduce postharvest weight loss and ethylene production in apples, while improving fruit firmness retention [87]. For highly perishable agricultural products, such as mushrooms, LEO treatment has been reported to improve browning-related parameters and delay quality deterioration [88]. Collectively, these findings indicate that LEO may contribute to food preservation not only through inhibition of spoilage-related microorganisms but also through regulation of certain postharvest physiological processes, thereby expanding its potential applications in the food industry.

### 3.3. Antiviral Activity of LEO

#### 3.3.1. Antiviral Mechanisms and Dual-Target Modulatory Effects of LEO

Viruses lack independent cellular structures, and their replication processes are highly dependent on host cells, often involving viral envelope fusion with host–cell membranes or receptor-mediated endocytosis. Based on these characteristics, the antiviral effects of LEO mainly involve two complementary mechanisms: direct targeting of viral structures and modulation of host immune responses.

**Targeting of Lipid Envelopes and Inhibition of Viral Entry:** The highly lipophilic monoterpenes abundant in LEO can directly interact with the lipid bilayer structures of enveloped viruses, including herpesviruses and coronaviruses [89]. By altering the integrity and stability of viral envelopes, LEO constituents may reduce the ability of viral particles to interact with host cells during the early stages of infection, thereby inhibiting viral attachment, membrane fusion, and subsequent entry into host cells.

**Host Immune Regulation and Inflammation Alleviation:** Viral infection and replication can induce host–cell damage and trigger dysregulated immune responses, including excessive release of inflammatory mediators and increased oxidative stress [90]. Due to its antioxidant and anti-inflammatory properties, LEO may regulate virus-associated inflammatory signaling pathways and attenuate tissue damage caused by excessive inflammatory responses, thereby providing potential auxiliary protective effects during viral infections [91].

#### 3.3.2. Inhibitory Effects of LEO Against Different Viral Strains

Herpes simplex virus type 1 (HSV-1) is a double-stranded DNA virus that causes localized infections and can establish latent infection within the host. Upon immune suppression, viral reactivation may occur, resulting in recurrent infections. Minami et al. reported that LEO derived from *L. latifolia* could effectively inhibit HSV-1 replication [92]. Further systematic screening demonstrated that stem extracts of *L. austroapennina* exhibited significant inhibitory activity against HSV-1 without inducing obvious cytotoxicity in Vero CCL-81 cells. In addition, these extracts showed inhibitory effects against human coronavirus 229E and non-enveloped poliovirus type 1 [89]. These findings suggest that volatile compounds derived from *Lavandula* species may exert antiviral effects by affecting viral structural stability or replication processes.

Highly pathogenic avian influenza H5N1 virus belongs to the influenza A virus group. Its hemagglutinin protein contains a multibasic cleavage site that can be activated by various host proteases, facilitating viral dissemination and inducing severe inflammatory responses and oxidative stress [93]. Studies have shown that LEO derived from *L. angustifolia* can alleviate H5N1 virus-induced inflammatory responses and oxidative stress [94]. This finding indicates that, in addition to potentially interfering with viral infection processes directly, LEO may exert auxiliary protective effects against virus-associated tissue damage through regulation of host inflammatory responses and oxidative stress pathways.

### 3.4. Anti-Inflammatory and Antioxidant Activities of LEO

Inflammation is a complex protective response triggered by harmful stimuli or tissue injury. This process involves coordinated regulation of various cytokines, transcription factors, inflammatory mediators, and pro-inflammatory genes, and it is typically characterized by clinical manifestations such as pain, redness, swelling, and impaired local function [95,96]. Under physiological conditions, moderate inflammatory responses are essential for pathogen elimination, tissue repair, and maintenance of homeostasis. However, persistent or excessive inflammatory activation may result in tissue damage and immune dysregulation and contribute to the development and progression of various chronic diseases.

Oxidative stress refers to a pathological condition caused by an imbalance between the production and elimination of free radicals, resulting in their excessive accumulation. Free radicals play important roles in normal metabolism and immune defense; however, excessive production can activate inflammatory signaling pathways. Conversely, sustained inflammatory responses further aggravate oxidative damage through the excessive generation of ROS [97]. Therefore, oxidative stress and inflammation interact reciprocally and amplify each other, forming a common pathological basis underlying various chronic diseases, including cancer, diabetes, cardiovascular diseases, Alzheimer’s disease, and osteoporosis [98].

When endogenous antioxidant and anti-inflammatory defense systems are insufficient to maintain physiological homeostasis, supplementation with appropriate exogenous bioactive compounds may play an important role in restoring oxidative–inflammatory balance [97]. LEO contains multiple bioactive constituents that can simultaneously regulate several key signaling pathways, thereby exerting multi-target effects on oxidative and inflammatory cascades.

#### 3.4.1. Core Molecular Mechanisms Underlying the Anti-Inflammatory and Antioxidant Activities of LEO

The anti-inflammatory and antioxidant effects of LEO and its major active constituents have been demonstrated in various experimental models. Modern molecular pharmacological studies indicate that the highly lipophilic volatile monoterpenes in LEO can readily penetrate cell membranes and rapidly modulate intracellular inflammatory and oxidative stress signaling pathways. Although individual constituents may regulate different molecular targets, their biological effects ultimately converge on several critical signaling axes, including NF-κB, MAPK, and Nrf2/HO-1 pathways. Across different inflammatory models and experimental systems, LEO has been shown to reduce the expression of inflammatory mediators and alleviate tissue damage. Representative findings are summarized in Table 5.

**Inhibition of NF-κB and MAPK Pro-Inflammatory Signaling Pathways:** LEO and its major constituents can effectively inhibit p65 phosphorylation and nuclear translocation, thereby suppressing NF-κB activation [105]. Mechanistic studies on isolated monoterpenes further illuminate these pathways. Specifically, 1,8-cineole can inhibit NF-κB/p65 signaling and significantly reduce the production of inflammatory cytokines, including TNF-α, IL-1β, and IL-6 [106]. Evidence from isolated terpinen-4-ol and α-terpineol (studied as tea tree oil constituents) demonstrates their ability to suppress LPS-induced inflammatory mediators in human macrophages via targeting NF-κB, p38 MAPK, and ERK MAPK signaling pathways [107]. Additionally, linalyl acetate (isolated from bitter orange leaf) exhibits selective inhibitory activity against COX-2 and PGE2 production [108]. While these isolated constituents reproduce key anti-inflammatory actions, their presence in whole LEO suggests potential multi-target engagement rather than a single-compound effect. Direct evidence from intact LEO supports these target interactions; for example, essential oil from L. angustifolia ‘Taikong blue’ attenuated LPS-induced responses in HaCaT and RAW264.7 cells through inhibition of the MAPK-NF-κB signaling network [109].

**Activation of Nrf2/HO-1 Antioxidant Defense Pathway:** Core constituents of LEO, particularly linalool, can activate the Nrf2/ARE signaling pathway and induce the expression of downstream cytoprotective proteins and endogenous antioxidant enzymes, including HO-1, SOD, CAT, and GPx. This process enhances cellular antioxidant defense capacity and reduces oxidative-stress-induced damage [110]. For example, pretreatment with *L. angustifolia* EO increased the levels of antioxidant enzymes, including SOD, GPx, and GSH, thereby protecting human hepatoma cells against H_2_O_2_-induced oxidative damage [8]. Giovannini et al. further demonstrated that *L. angustifolia* EO could restore S. aureus-induced inflammatory signaling abnormalities by suppressing major pro-inflammatory cytokines and promoting HO-1 gene transcription [101].

#### 3.4.2. Multi-Organ Protective Effects of LEO in In Vivo Models of Inflammation and Oxidative Stress

Based on the aforementioned molecular mechanisms, LEO has demonstrated broad protective effects in peripheral tissues, metabolic disorders, and the central nervous system through regulation of inflammatory responses and oxidative stress pathways.

**Local Acute Inflammation and Peripheral Edema:** In classical inflammatory models, *L. angustifolia* EO has been shown to effectively alleviate carrageenan-induced paw edema, croton oil-induced pleurisy, and TPA-induced ear inflammation in mice [99,100,102]. These effects are associated with significant downregulation of inflammatory mediators, including TNF-α and COX-2, in local tissues (Figure 2B). Notably, its inhibitory effect on edema formation was comparable to that of the standard anti-inflammatory drug dexamethasone [103].

**Metabolic Disorders and Target Organ Protection:** In systemic metabolic disorders, LEO protects vulnerable organs through its anti-inflammatory and antioxidant properties. For example, in an alloxan-induced diabetic rat model, a combination of *L. stoechas* EO and rosemary essential oil significantly reduced the levels of oxidative stress markers, including malondialdehyde (MDA) and H_2_O_2_, while enhancing the activities of antioxidant enzymes such as SOD, CAT, and GPx in the testes and epididymis of diabetic male rats, thereby alleviating diabetes-associated reproductive damage [111]. In response to chemical toxicants and metabolic by-products, both *L. stoechas* EO and *L. officinalis* EO have been reported to alleviate hepatic and renal oxidative damage induced by malathion and exogenous H_2_O_2_ in mice. These protective effects were associated with reduced MDA and H_2_O_2_ levels and enhanced activities of antioxidant enzymes, including SOD, CAT, and GPx [112,113]. In addition, *L. latifolia* EO has been shown to alleviate hyperuricemia-associated inflammation in rat models by modulating arachidonic acid metabolism and suppressing NF-κB signaling pathways [114].

**Neuroprotection and Maintenance of Cognitive Function:** Through its antioxidant and anti-inflammatory activities, LEO exerts neuroprotective effects in various in vivo models. Regarding neuroinflammatory regulation, *L. dentata* EO has been reported to modulate ion channels and electrolyte balance, reduce neuronal excitability, and restore the balance of Treg- and Th17-associated cytokines. These effects normalize pro-inflammatory and anti-inflammatory cytokine profiles, as well as Treg-related markers, thereby improving histopathological alterations and delaying epileptogenesis in male Wistar rats [115]. In a mouse cerebral ischemia/reperfusion model, *L. angustifolia* EO reduced MDA and ROS levels while increasing SOD, CAT, and GSH-Px activities, as well as the GSH/GSSG ratio, thereby attenuating oxidative stress-mediated neuronal injury [116]. At the temporal lobe tissue level, different subspecies of *L. angustifolia* EO significantly enhanced SOD, GPx, and CAT activities while reducing total GSH and MDA levels in rat temporal lobe homogenates, suggesting that its neuroprotective effects are primarily associated with antioxidant mechanisms [117]. Furthermore, LEO has been shown to alleviate cerebral ischemic injury and improve cognitive function in Alzheimer’s disease models through enhancement of endogenous antioxidant defenses, suppression of inflammatory responses, and regulation of acetylcholinesterase activity [118,119,120]. Collectively, these protective effects are closely associated with activation of the Nrf2/HO-1 antioxidant pathway and inhibition of NF-κB-mediated inflammatory signaling, as described in the previous section.

#### 3.4.3. Factors Affecting the Antioxidant Activity of LEO and Its Industrial Application Potential

The antioxidant activity of LEO is influenced by multiple factors, including *Lavandula* species, cultivars, extraction methods, and environmental conditions. Currently, the antioxidant capacity of LEO is mainly evaluated using various in vitro assays, such as DPPH, ABTS, and TEAC methods. However, differences in analytical protocols and reference standards among studies have resulted in considerable variation in reported antioxidant activities (Table 6).

Significant differences in antioxidant activity have been observed among different *Lavandula* species and cultivars. Based on DPPH and ABTS assays, Carrasco et al. demonstrated that *L. angustifolia* EO exhibited stronger antioxidant activity than *L. hybrida* cv. ‘Super’, which was further confirmed by oxygen radical absorbance capacity (ORAC) analyses [19]. In contrast, Blažeković et al. reported that *L. × intermedia* ‘Budrovka’ EO showed significantly higher DPPH radical-scavenging activity than *L. angustifolia* EO, and this activity was not primarily determined by the most abundant constituents, such as linalool or linalyl acetate [122]. Among five *L. stoechas* cultivars cultivated in Thailand, *L. stoechas* × viridis ‘St. Brelade’ EO exhibited the strongest antioxidant activity in both DPPH and ABTS assays, which may be attributed to differences in chemical composition among cultivars [4].

Extraction technology represents another critical factor affecting the antioxidant properties of LEO. Danh et al. reported that LEO obtained by supercritical carbon dioxide extraction (SCE) exhibited significantly higher antioxidant activity than those obtained by steam distillation and hexane extraction. This enhanced activity may be partially attributed to the relatively mild extraction conditions of SCE, which is performed at 45 °C and can better preserve the native properties of bioactive constituents [10].

Cultivation conditions and plant tissues also contribute to variations in antioxidant activity. Andrys et al. evaluated the antioxidant capacities of three *L. angustifolia* EOs and found that EOs obtained from in vitro cultured plants exhibited significantly higher antioxidant activity than those from field-grown plants, as demonstrated by DPPH, ABTS, and fluorescence recovery after photobleaching assays [121]. Furthermore, Smigielski et al. reported that, within the same plant, EO extracted from fresh aerial parts of *L. angustifolia* exhibited the strongest antioxidant activity [123].

Beyond its biological protective effects, the antioxidant activity of LEO also demonstrates considerable potential for industrial applications, particularly in the food processing industry. Lipid oxidation is one of the major causes of food quality deterioration, and the addition of antioxidants can effectively prevent or delay this process. Studies have shown that *L. latifolia* EO can inhibit the oxidation of soybean edible oil more effectively than untreated control oil, significantly reducing the loss of unsaturated fatty acids and improving oxidative stability, indicating its potential as a natural antioxidant stabilizer [124]. Furthermore, Bulai et al. evaluated the effects of LEO on the physicochemical and textural properties of smoked sausages, providing additional evidence for its potential application in meat preservation [125]. Collectively, these findings demonstrate that the antioxidant activity of LEO not only provides biological protective benefits but also highlights its promising potential as a natural antioxidant additive in the food industry.

### 3.5. Multi-Target Regulatory Effects of LEO on the Central and Peripheral Nervous Systems

The pathophysiological mechanisms underlying neurological disorders are complex and involve multiple factors, including neurotransmitter imbalance, ion channel dysfunction, and neuroinflammation. Current evidence suggests that the neuroregulatory effects of LEO are primarily mediated through two complementary pathways. First, highly volatile aromatic compounds in LEO can directly activate the limbic system through olfactory pathways, thereby inducing psychological relaxation and emotional regulation responses. Second, the major small-molecule monoterpenes in LEO, particularly linalool and linalyl acetate, can rapidly penetrate the blood–brain barrier and interact with specific molecular targets in both the central and peripheral nervous systems.

These bioactive constituents can modulate multiple key G protein-coupled receptors (GPCRs) and ion channels involved in neural regulation rather than acting on a single target. This multi-target synergistic mechanism enables LEO to simultaneously regulate neurotransmitter signaling imbalance, ionic homeostasis disruption, and neuroinflammatory responses, thereby exhibiting broad regulatory potential in mood disorders, seizures, pain, and neurodegenerative diseases [126,127]. The following sections will provide an in-depth analysis of the neuropharmacological effects of LEO based on its major molecular target families.

#### 3.5.1. LEO Improves Mood Disorders and Sleep Disorders Through Modulation of GABA_A_, NMDA, and 5-HT_1A_ Receptors

The functional stability of the central nervous system (CNS) largely depends on the dynamic balance between inhibitory (GABAergic) and excitatory (glutamatergic) neurotransmission. LEO and its major monoterpene constituents are capable of modulating this balance through allosteric regulation of key neurotransmitter receptors, which represents a central molecular mechanism underlying their anxiolytic, antidepressant, and sedative-hypnotic effects.

**Activation of GABA_A_ Receptors to Enhance Inhibitory Neurotransmission:** Regarding inhibitory synaptic transmission, studies using pure compounds show that linalool acts as a positive allosteric modulator of GABA_A_ receptors. Studies using Xenopus laevis oocytes demonstrated that isolated linalool exerts sedative effects through allosteric modulation of the GABA_A_ receptor α1β2γ2 subtype [128]. Further investigations revealed that the metabolites of linalool may also contribute to GABAergic modulation. For example, oxygenated linalool metabolites at the C8 position were shown to positively regulate GABAergic currents, thereby influencing inhibitory neurotransmission [129]. These findings suggest that the in vivo effects of LEO may result from the combined and sustained actions of the parent compounds and their bioactive metabolites [129].

**Antagonism of NMDA Receptors to Prevent Excitotoxicity:** As ionotropic glutamate receptors, NMDA receptors play essential roles in excitatory synaptic transmission in the CNS. However, excessive NMDA receptor activation is closely associated with neuronal excitotoxicity, epilepsy, and neurodegenerative disorders. At the receptor level, isolated monoterpenes, particularly linalool and linalyl acetate, have been reported to interact with NMDA receptors, contributing to neuroprotective and anxiolytic-like effects [130,131]. In addition, isolated linalool may suppress glutamate release induced by nociceptive somatic stimulation, thereby indirectly modulating NMDA receptor-mediated excitatory signaling pathways [132].

**Activation of 5-HT_1A_ Receptors and Inhibition of SERT:** Regarding antidepressant activity, isolated linalool in LEO may enhance serotonergic neurotransmission through multiple mechanisms. It can inhibit monoamine oxidase A (MAO-A) activity and reduce serotonin transporter (SERT)-mediated reuptake at presynaptic membranes, leading to increased serotonin (5-HT) availability within the synaptic cleft [131,133,134]. Elevated 5-HT levels subsequently activate postsynaptic 5-HT_1A_ receptors, thereby enhancing serotonergic signaling and producing antidepressant effects in the forced swimming test model (Figure 2C) [131,132,133,134].

Based on these multi-receptor regulatory mechanisms, clinical studies have demonstrated that inhalation of LEO, particularly *L. angustifolia* EO, may represent an effective, safe, and feasible complementary intervention for reducing anxiety and depressive symptoms in various populations, including patients undergoing surgery, individuals with cardiovascular diseases, patients receiving electroconvulsive therapy or hemodialysis, as well as children and older adults [135,136,137,138]. Inhalation of *L. angustifolia* EO has also been shown to attenuate physiological stress responses and facilitate recovery after stress exposure [139]. Lavender aromatherapy patches applied to women undergoing breast surgery have been reported to alleviate perioperative anxiety [140]. Bridging the gap between these single-compound target mechanisms and whole-plant clinical applications, oral administration of the standardized lavender preparation Silexan™ (derived from *L. angustifolia* flowers) has been demonstrated in multiple clinical trials to significantly improve mild-to-moderate anxiety, mixed anxiety-depressive symptoms, and associated sleep disturbances. Importantly, these therapeutic effects were achieved without significant adverse effects on cognitive or motor functions [141].

#### 3.5.2. LEO Targets TRPA1 Channels and the Catecholaminergic System to Alleviate Pain and Neural Hypersensitivity

In addition to its effects on mood and cognitive disorders, LEO has demonstrated considerable potential in the management of complex pain syndromes and neurological hypersensitivity conditions, including seizures and migraine. Based on neurobiological mechanisms, pain can be classified into nociceptive pain, inflammatory pain, and neuropathic pain [142,143,144]. Through its major bioactive constituents, LEO may exert analgesic effects by regulating specific ion channels, suppressing inflammatory amplification cascades, and modulating pain signaling at both peripheral and spinal levels.

**Selective Inhibition of the TRPA1 Ion Channel:** The transient receptor potential ankyrin 1 (TRPA1) channel is a critical peripheral nociceptive receptor involved in pain signal transduction. Recent studies focusing on isolated single constituents have demonstrated that linalyl acetate can selectively inhibit TRPA1 channels expressed in nociceptive sensory neurons, thereby directly suppressing pain-related neuronal signaling and alleviating nociceptive and inflammatory pain [145]. Similarly, isolated camphor has been shown to selectively inhibit TRPA1 activity and reduce abnormal high-frequency action potential firing in dorsal root ganglion (DRG) nociceptive neurons, thereby attenuating neuropathic pain induced by chemotherapy or tissue injury (Figure 2D) [146].

**Regulation of the Catecholaminergic System and COX-2 Pathway:** Preliminary findings suggest that, at the spinal level, isolated linalool may engage the catecholaminergic system and selectively modulate α_2_-adrenergic, β_2_-adrenergic, and D_2_/D_3_ dopamine receptors, which could contribute to inhibiting the central transmission of nociceptive signals and attenuating neuropathic pain in diabetic models [147]; however, these underlying spinal pathways warrant further rigorous peer-reviewed validation. In inflammatory pain models, isolated linalyl acetate exerts anti-inflammatory analgesic effects through selective inhibition of cyclooxygenase-2 (COX-2), resulting in reduced prostaglandin E_2_ (PGE_2_) production and suppression of inflammatory signal amplification [108].

**Regulation of Smooth Muscle Activity and Vascular Tone:** Beyond direct modulation of pain pathways, LEO constituents may influence neural hypersensitivity through regulation of ion channels, smooth muscle function, and vascular responses. Isolated linalool exhibits anticonvulsant activity by regulating potassium and sodium currents and enhancing GABAergic inhibitory signaling [148]. Meanwhile, isolated linalyl acetate may reduce migraine severity by suppressing neurogenic inflammation and regulating vascular tone and smooth muscle function, thereby decreasing the frequency and intensity of migraine attacks [149].

Translating these constituent-level mechanisms to clinical practice with whole LEO, clinical studies have demonstrated that LEO administration through aromatherapy inhalation or topical massage can significantly reduce pain intensity in patients with burns, knee osteoarthritis, pediatric tooth extraction, and postoperative conditions, while improving overall quality of life [150,151]. In addition, self-administered abdominal aromatherapy massage with LEO has been shown to effectively relieve menstrual pain [152]. For postherpetic neuralgia, a chronic neuropathic pain condition that severely affects quality of life, LEO and its major active constituents may reduce pain intensity and improve patients’ overall health status through multimodal analgesic mechanisms [153].

### 3.6. LEO Promotes Wound Healing and Skin Barrier Repair Through Multi-Target Synergistic Actions

As the largest organ of the human body, the skin serves as a critical barrier protecting against external stimuli. Impairment of skin barrier function, such as that caused by trauma, ulcers, dermatitis, or microbial infection, can disrupt local immune and metabolic homeostasis within the wound microenvironment. Studies have demonstrated that the highly lipophilic constituents of LEO can penetrate the stratum corneum and reach effective concentrations at damaged skin sites. The beneficial effects of LEO on skin barrier restoration are not merely attributable to physical protection but are primarily mediated through activation of local receptors, regulation of growth factor expression, and coordinated antibacterial and anti-inflammatory activities [154,155].

#### 3.6.1. Core Molecular Mechanisms Underlying LEO-Mediated Wound Healing and Skin Barrier Repair

**Targeting Activation of Ectopically Expressed Olfactory Receptors in the Skin:** Recent studies have revealed that human skin cells ectopically express multiple olfactory receptors, which participate extensively in cutaneous physiological regulation and metabolic processes [156,157]. In vitro receptor ligand studies demonstrate that specific isolated monoterpenes present in LEO function as active ligands for these receptors [156,157]. In particular, isolated linalool has been shown to selectively activate the olfactory receptors OR2AG2 and OR10A6 expressed in skin tissues, thereby participating in the regulation of local inflammatory responses and oxidative stress signaling cascades [156]. In addition, isolated acetate-derived constituents (such as linalyl acetate) may activate the OR51E2 receptor and significantly inhibit melanocyte proliferation, providing a potential molecular basis for their application in pigmentation regulation and related dermatological disorders [157]. While these receptor pathways were characterized using pure compounds, they uncover a novel target domain for intact LEO in skin barrier repair.

**Activation of growth factor expression and extracellular matrix remodeling:** During tissue regeneration, LEO can directly stimulate fibroblast proliferation and differentiation, thereby promoting collagen synthesis and accelerating wound closure [154]. Mechanistic studies indicate that the wound-healing effects of LEO are closely associated with upregulation of interleukin-6 (IL-6), a cytokine recognized as an important mediator initiating tissue repair responses [16]. In addition, this process is accompanied by increased expression of vascular endothelial growth factor (VEGF), platelet-derived growth factor (PDGF), and epidermal growth factor (EGF). Notably, transforming growth factor-β (TGF-β) signaling plays a central regulatory role during this process. LEO-induced upregulation of TGF-β promotes fibroblast proliferation and differentiation into contractile myofibroblasts, thereby accelerating wound contraction, granulation tissue formation, and collagen deposition [154,158]. The subsequent release of these mitogenic factors facilitates angiogenesis and extracellular matrix remodeling, ultimately improving the structural integrity and mechanical strength of regenerated tissue [16,159].

#### 3.6.2. In Vivo Evidence of LEO-Mediated Wound Healing and Tissue Regeneration and Optimization of Delivery Strategies

In preclinical models, the receptor-mediated regulation and growth factor activation mechanisms described above have been effectively translated into measurable wound repair outcomes. In acute excisional wound and burn models, topical application of LEO-containing formulations has been shown to promote wound edge contraction and accelerate the re-epithelialization process [160,161]. As discussed previously, microbial colonization within the wound microenvironment represents a major barrier to tissue regeneration. Combined with the potent antimicrobial properties of LEO (see Section 3.1), its application in wound management provides a dual benefit by integrating microbial control and tissue regeneration. A systematic review indicated that LEO represents a promising candidate for topical wound-care formulations, as it can not only reduce pathogen colonization in open wounds and decrease the risk of surgical site infections but also promote granulation tissue contraction, thereby lowering the likelihood of wound suppuration and delayed healing [158]. For chronic non-healing wounds associated with microvascular impairment and immune dysfunction, such as diabetic ulcers, LEO may accelerate wound closure through the regulation of key growth factors and inflammatory mediators involved in tissue repair [154].

At the translational level, these preclinical mechanisms have been further supported by randomized controlled clinical trials investigating human surgical wound healing. In particular, episiotomy wounds after childbirth represent a common model of acute surgical skin injury. Topical administration of LEO-containing preparations has demonstrated beneficial effects on tissue repair. Clinical evaluations showed that LEO interventions significantly reduced REEDA scores, which assess redness, edema, ecchymosis, discharge, and wound approximation, while promoting early wound closure, alleviating local swelling, and providing measurable analgesic effects [158,162,163]. This successful translation from molecular mechanisms, including growth factor induction and cellular regulation, to improved clinical wound-healing outcomes highlights the promising application potential of LEO in advanced wound-care management.

However, essential oil generally possesses inherent physicochemical limitations, including high volatility and susceptibility to oxidation. When directly applied as a single topical treatment to open wounds, maintaining effective local concentrations over extended periods can be challenging. To overcome these limitations and improve LEO stability and controlled release efficiency, various advanced delivery systems have been developed, including LEO-loaded polyvinyl alcohol–lysine electrospun membranes and nanoemulsion-based gels. These sustained-release wound dressings can encapsulate volatile components, enable prolonged release of active compounds at wound sites, maintain long-lasting antimicrobial effects, and further promote continuous fibroblast migration, demonstrating substantial potential as advanced wound-healing materials [164,165].

#### 3.6.3. Symptom Control and Skin Homeostasis Regulation by LEO in Inflammatory Skin Disorders

Beyond its role in physical wound repair, LEO has also demonstrated promising pharmacological effects in the management of inflammatory skin disorders, including psoriasis, atopic dermatitis, and acne, through modulation of inflammatory responses, microbial balance, and skin barrier function.

In psoriasis, studies have shown that linalool and linalyl acetate, two major constituents of LEO, can significantly alleviate psoriasis-associated histopathological alterations. Specifically, linalool primarily exerts its effects by downregulating IL-17 and IL-22 levels, whereas linalyl acetate markedly suppresses the expression of Th1-associated inflammatory cytokines [16,166]. These findings suggest that LEO components may interfere with key immune pathways involved in psoriasis pathogenesis, particularly those associated with Th17/Th1-mediated inflammatory responses. In a canine model of DNCB-induced allergic contact dermatitis, a topical aromatic oil formulation containing lavender, Roman chamomile, and tea tree oils significantly reduced transepidermal water loss (TEWL) and erythema scores. Meanwhile, it suppressed excessive expression of pro-inflammatory cytokines, such as TNF-α, in dermal tissues, thereby promoting restoration of the impaired skin barrier [167]. Furthermore, studies utilizing the NC/Nga mouse model of atopic dermatitis demonstrated that topical application of lavender-containing essential oil mixtures (e.g., combined with German chamomile and sandalwood or with lemon and eucalyptus) significantly attenuated serum IgE and IgG1 levels, downregulated Th2-related inflammatory cytokines (such as IL-4 and IL-13), and accelerated lesion recovery [168,169]. These findings indicate that LEO-containing formulations may regulate both immune hypersensitivity and epidermal barrier dysfunction, two central pathological features of atopic dermatitis.

In clinical acne management, a double-blind, placebo-controlled study demonstrated that the topical application of a formulation containing lavender essential oil (combined with tea tree, eucalyptus, and tangerine oils) significantly reduced non-inflammatory acne lesions and decreased comedone counts after 90 days. Furthermore, non-invasive imaging revealed a marked improvement in follicular hyperkeratinization and a reduction in pilosebaceous unit size, which can be attributed to the synergistic anti-inflammatory, keratinization-normalizing, and antibacterial properties of the terpene components [170].

### 3.7. Other Biological Activities of LEO

In addition to its well-characterized effects on local microenvironment regulation and central nervous system modulation, LEO has demonstrated broad biological activities in systemic organ protection, tumor suppression, and defense against exogenous pathogens. These diverse biological effects further highlight its translational potential as a multi-target natural compound.

#### 3.7.1. Targeting Inflammatory–Apoptotic Cascades and Systemic Organ Protection

**Alleviation of Intestinal and Airway Immune Dysregulation:** In a mouse model of ulcerative colitis driven by epithelial barrier disruption and immune imbalance, *L. × intermedia* EO significantly reduced the expression of key inflammatory mediators, including TNF-α, IFN-γ, IL-22, and MIP-2α. Meanwhile, it inhibited neutrophil and macrophage infiltration, thereby alleviating colonic tissue injury [104,171]. In respiratory inflammatory disorders, LEO has also demonstrated potential protective effects. In an ovalbumin-induced asthma mouse model, LEO significantly decreased IL-5 and IL-13 levels in bronchoalveolar lavage fluid, reduced pulmonary expression of IL-4 and IL-5, and downregulated Muc5b mRNA expression in lung tissues. These effects were associated with reduced airway inflammation, mucus hypersecretion, and perivascular eosinophil infiltration, suggesting potential value as an adjunctive intervention for bronchial asthma [172].

**Regulation of Cell Proliferation and Cardiovascular Protection:** Across cardiovascular protection and cancer-related research, LEO exhibits a common regulatory pattern involving modulation of the balance between cellular proliferation and apoptosis. In a myocardial infarction model established in male Wistar rats, LEO alleviated myocardial injury through antioxidant and anti-apoptotic mechanisms, restoring the reduced expression of anti-apoptotic factors and vascular endothelial growth factor (VEGF) associated with cardiac damage [173]. Furthermore, in a subacute administration model of pulmonary thromboembolism in mice, LEO significantly reduced thrombotic events without inducing hemorrhagic complications, suggesting potential antithrombotic activity with an acceptable safety profile [174]. In emerging oncology research, *L. angustifolia* EO and its major component linalool have been shown to significantly inhibit the proliferation of PC-3 prostate cancer cells. This inhibitory effect was accompanied by downregulation of proliferation markers, including Ki67 and PCNA, together with induction of apoptosis [175]. Moreover, this EO did not exhibit mutagenic toxicity in mouse splenocytes, suggesting potential dual activities involving anticancer effects and protection against genotoxic damage [176].

#### 3.7.2. Broad-Spectrum Antiparasitic Activity and Agricultural Pest Management Applications

**Medical Parasitic Disease Control:** *L. stoechas* EO has been demonstrated to possess potent antiparasitic activity against several Leishmania species, including Leishmania major, *L. tropica*, and *L. infantum* [177] (Table 7). Further in vitro investigations have shown that LEO can inhibit the invasion and proliferation of *Toxoplasma gondii* by disrupting parasite membrane integrity, highlighting its potential as a candidate for the development of anti-Toxoplasma agents [16]. In addition, in vitro parasitic models demonstrated that LEO exhibited acaricidal and pediculicidal activities, which were even stronger than those of lemon essential oil at the same concentration [178].

**Agricultural Pest Management:** Recent studies by Awad et al. demonstrated that *L. multifida* EO could serve as an environmentally friendly botanical insecticide for controlling destructive agricultural pests, including the Egyptian cotton leafworm (*Spodoptera littoralis*) and the black cutworm (*Agrotis ipsilon*) [182]. These findings not only expand the potential applications of LEO beyond biomedical fields but also provide scientific support for its future development in sustainable agriculture and integrated pest management strategies.

## 4. Conclusions and Future Perspectives

### 4.1. Limitations of Current Evidence

While accumulating research highlights the diverse pharmacological potential of LEO, a critical and objective evaluation reveals several methodology- and evidence-level limitations that warrant caution when interpreting current findings [18]:

**Predominance of Pre-Clinical and Single-Constituent Evidence:** A substantial proportion of reported mechanisms, particularly those involving specific receptor targets (e.g., TRPA1, OR2AG2) and intracellular pathways (e.g., NF-κB, Nrf2/HO-1), are derived from in vitro assays or animal models using isolated single monoterpenes (e.g., pure linalool or linalyl acetate) [108,128,145,156]. Direct mechanistic validation utilizing whole, intact LEO in complex physiological systems remains relatively limited [100].

**Imbalance and Narrow Scope in Clinical Validation:** High-level clinical evidence is overwhelmingly concentrated on a single, highly standardized oral formulation—Silexan™—predominantly evaluated for anxiety and sleep disorders [141]. In contrast, clinical evidence supporting other widely claimed therapeutic applications, such as systemic anti-inflammatory, tissue repair, and broad antimicrobial efficacy, remains sparse, largely anecdotal, or constrained by small sample sizes and non-rigorous controls [16,158].

**Chemical Heterogeneity and Publication Bias:** The high compositional variability driven by genetics, extraction methods, and environmental factors implies that therapeutic claims are strictly chemotype-specific rather than universally applicable to all “lavender oils” [19,21,22]. Furthermore, the field suffers from a potential publication bias, where negative or null results are rarely reported, and LEO preparations occasionally fail to demonstrate statistically significant superiority or equivalence when benchmarked against standard reference drugs [103].

Addressing these evidence gaps through standardized reporting, well-designed multi-center clinical trials, and rigorous comparative pharmacology will be essential to advance LEO from traditional empirical use to evidence-based medicine [18].

### 4.2. Future Perspectives and Translational Strategies

As a classical natural plant-derived product, LEO has demonstrated pharmacological potential far beyond its traditional applications in aromatherapy. This review systematically summarizes the biological activities and underlying molecular mechanisms of LEO across multiple fields, including neuromodulation, skin barrier repair, antimicrobial defense, and systemic organ protection. Current evidence indicates that the pharmacological effects of LEO do not result from a single constituent but rather from the highly coordinated interactions between major volatile monoterpenes, particularly linalool and linalyl acetate, together with diverse minor bioactive compounds. However, several translational challenges remain, and future research should focus on the following key directions:

**Transition from Single Essential Oil Extraction Toward a “Dual Pharmacological System” and Whole-Plant Valorization:** Current industrial and academic efforts have largely focused on volatile essential oil, resulting in extensive waste of post-distillation plant residues that remain rich in bioactive non-volatile compounds, including polyphenols, flavonoids, and rosmarinic acid with antioxidant and anti-inflammatory properties. Future strategies should move beyond the single-product paradigm and consider lavender as a dual pharmacological system consisting of volatile essential oil components and non-volatile bioactive biomass. Through the development of advanced green extraction technologies, these residual materials can be further valorized, enabling exploration of their potential roles in chronic inflammatory disorders and neurodegenerative diseases while aligning with the global transition toward a circular bioeconomy [183].

**Advancement of Clinical Translation and Establishment of Characteristic Chemotype-Based Standardization Systems:** Although standardized LEO preparations, such as Silexan™, have achieved notable clinical success in the management of anxiety disorders, evidence supporting its applications in anti-inflammatory, anticancer, and peripheral organ-protective effects remains largely limited to in vitro and animal studies [18]. Given that LEO chemical profiles are highly influenced by geographic origin, altitude, climatic variation, and extraction processes, future studies should include larger-scale, multicenter randomized controlled clinical trials. In parallel, the establishment of standardized chemical fingerprinting systems linking specific chemotypes (e.g., linalool/linalyl acetate type and 1,8-cineole type) with corresponding pharmacological activities will be essential for reducing batch-to-batch variability and ensuring reproducible therapeutic efficacy.

**Overcoming Physicochemical Limitations Through Advanced Intelligent Delivery Systems:** Due to the inherent limitations of essential oils, including high volatility, poor aqueous solubility, susceptibility to oxidative degradation, and potential skin sensitization at elevated concentrations, future formulation research should adopt interdisciplinary approaches. The development of advanced delivery platforms, such as liposomes, nanoemulsion-based gels, electrospun membranes, and smart-responsive carriers, may substantially improve the local bioavailability and sustained-release capacity of LEO active components at target sites, including open wounds and inflammatory microenvironments. Moreover, these systems may help reduce potential contact toxicity and enhance the safety profile of LEO-based products [16,18].

In conclusion, with the advancement of multidisciplinary integration, lavender research is undergoing a profound paradigm shift from traditional empirical applications toward evidence-based molecular pharmacology. Fully elucidating the complexity of its chemical composition and multi-target regulatory networks, while accelerating the transition from laboratory investigations to clinical applications, will provide new opportunities for the development of effective and safe natural therapeutics and modern health-oriented products.

## Figures and Tables

**Table 1:** Main chemical composition (%) of lavender essential oil from different varieties, geographical origins, plant materials, and extraction methods.

Component	LDE	LST	LMU	LSS	LSW	LSM	LSA	LSVS	LAM–B	LAM–R	LAM–M	LCO-F	LCO-L	LCO-W	LAL-H	LAL-X	LAL-S
Origin	Tunisia			Thailand					Budva	Rovinj	Montenegro	Jordan			Australia		
Plant Part	Aerial parts			Aerial parts					Flowers			Flowering tops	Leaves	Whole aerial parts	Flowers		
Extraction Method	Hydrodistillation			Hydrodistillation					Hydrodistillation			Hydrodistillation			Hydrodistillation	Hexane extraction	SCE
Reference	[22]			[4]					[23]			[24]			[10]		
Linalool	47.3	20.25	50.05	–	–	–	–	–	27.32	47.67	19.92	41.16	40.78	20.77	52.59	33.35	42.82
Linalyl acetate	28.65	64.3	7.3	–	–	–	–	–	1.5	4.56	9.73	–	–	–	9.27	25.73	23.4
β–Thujone	0.02	8.97	0.14	–	–	–	–	–	–	–	–	–	–	–	–	–	–
Camphene	0.01	0.33	10.06	0.6	1.1	4.78	–	1.27	0.7	0.43	0.64	0.17	0.19	0.29	0.19	–	–
1,8–Cineole	0.44	0.04	0.19	13.3	23.89	9.73	7.83	33.86	–	9.6	14.89	7.31	7.72	25.43	1.51	0.55	1.18
Fenchone	tr	0.03	0.2	35.42	37.18	23.73	23.24	30.94	–	–	–	–	–	–	–	–	–
Camphor	2.32	0.04	0.3	28.82	3.68	35.3	42.47	7.13	7.52	11.82	21.23	11.57	12.1	19.71	8.79	6.81	8.05
β–Phellandrene	–	–	–	–	–	–	–	–	19.1	0.16	–	–	–	–	–	–	–
Borneol	0.07	0.07	0.21	–	–	–	–	–	20.24	8.11	5.33	6.76	7.41	5.59	7.5	7.5	6.68
Terpinen–4–ol	0.82	0.09	0.24	0.58	–	–	–	–	4.6	3.08	0.74	10.97	11.35	4.93	2.45	2.12	2.16
α–Terpineol	0.67	0.97	0.26	–	0.98	0.47	–	1.28	0.85	1.86	2.01	1.03	0.79	1.01	3.03	0.8	0.73
α–Pinene	0.06	0.96	0.45	1.2	4.89	4.75	3.13	4.33	0.72	0.35	0.26	0.38	0.45	0.56	–	–	–
Terpinolene	0.04	0.03	2.05	–	3.23	–	–	4.99	0.37	0.05	–	0.54	0.66	0.23	0.21	–	–
Total	76.8	96.8	71.45	79.92	74.95	78.76	76.67	83.8	82.92	87.69	74.75	79.89	81.45	78.52	85.54	76.86	85.02

Notes: tr indicates traces (<0.01%); – indicates not determined or not reported. Abbreviations: LDE: *Lavandula dentata*; LST: *L. stoechas*; LMU: *L. multifida*; LSS: *L. stoechas*‘snowman’; LSW: *L. stoechas*‘white lavender’; LSM: *L. stoechas*‘major’; LSA: *L. stoechas*‘avonview’; LSVS: *L. stoechas* × *viridis*‘St. Brelade’; LAM-B: *L. angustifolia* Mill. from Budva; LAM-R: *L. angustifolia* Mill. from Rovinj; LAM-M: *L. angustifolia* Mill. from Montenegro; LCO-F: *L. coronopifolia* Flowering tops; LCO-L: *L. coronopifolia* leaves; LCO-W: *L. coronopifolia* whole aerial parts; LAL-H: *L. angustifolia* L. (hydrodistillation); LAL-X: *L. angustifolia* L. (hexane extraction); LAL-S: *L. angustifolia* L. (supercritical CO_2_ extraction); SCE: supercritical CO_2_ extraction.

**Table 2:** Main chemical composition ranges (%) of Lavandula angustifolia Mill. essential oil according to ISO 3515:2002.

Component	Spontaneous Lavender	Clonal Lavenders (Principal Origins)				
	France	France “Maillette”	Bulgaria	Russian Federation	Australia	Other Origins
Limonene	≤0.5	≤0.3	≤0.6	≤1	≤0.5	≤1
1,8-Cineole	≤1	≤0.5	≤2	≤2.5	≤1	≤3
β-Phellandrene	tr~0.5	≤0.2	≤0.6	≤1	≤0.5	≤1
cis-β-Ocimene	4~10	≤2.5	3~9	3~8	3~9	1~10
trans-β-Ocimene	1.5~6	≤2	2~5	2~5	0.5~1	0.5~6
3-Octanone	tr~2	1~2.5	0.2~1.6	≤0.6	2~5	≤3
Camphor	tr~0.5	≤1.2	≤0.6	≤0.6	≤0.5	≤1.5
Linalool	25~38	30~45	22~34	20~35	25~38	20~43
Linalyl acetate	25~45	33~46	30~42	29~44	25~45	25~47
Lavandulol	≥0.3	≤0.5	≥0.3	≥0.1	≥0.3	≤3
Terpinen-4-ol	2~6	≤1.5	2~5	1.2~5	1.5~6	≤8
Lavandulyl acetate	≥2	≤1.3	2~5	1~3.5	≥1	≤8
α-Terpineol	≤1	0.5~1.5	0.8~2	0.5~2	≤1	≤2

Notes: tr indicates traces (<0.01%). Abbreviations: ISO: International Organization for Standardization.

**Table 3:** Antibacterial activity of lavender essential oil against different bacterial strains.

Lavender Source	Bacteria	MIC	MBC	Original Unit	Normalized MIC/MBC (mg/mL)	Proposed Targets/Modes of Action	Study Limitations	Reference
Lavender (Poland)	*Staphylococcus aureus*	3.0	4.25	μL/mL	2.67/3.78	–	In vitro disc diffusion and broth microdilution; mechanism not experimentally evaluated	[50]
	*Enterococcus faecalis*	2.75	4.0		2.45/3.56			
	*Escherichia coli*	3.5	4.5		3.12/4.01			
	*Klebsiella pneumoniae*	4.0	5.0		3.56/4.45			
	*Enterobacter cloacae*	4.0	5.0		3.56/4.45			
	*Acinetobacter baumannii*	4.5	5.25		4.01/4.67			
*L. angustifolia* Mill.	Methicillin–resistant *Staphylococcus aureus* (ATCC 43300)	18.29	439	mg/mL	18.29/439	Cell wall carbohydrate/phosphate structure alteration and cell membrane permeabilization (confirmed by FTIR)	In vitro assay only; high concentrations required for single essential oil	[51]
*L. maroccana* Murb.	*S. aureus* (CCMMB3)	4.604	4.604	mg/mL	4.604/4.604	Disruption of plasma membrane integrity, dissolution of proton motive force, and inhibition of ATP synthesis	In vitro screening study; specific downstream intracellular signaling pathways not investigated	[52]
	*Micrococcus luteus* (ATCC 10,240)	2.302	2.302		2.302/2.302			
	*Bacillus subtilis* (ATCC 9524)	2.302	2.302		2.302/2.302			
	*E. coli* (ATCC 8739)	9.208	9.208		9.208/9.208			
	*K. pneumoniae*	4.604	9.208		4.604/9.208			
	*Pseudomonas aeruginosa* (DSM, 50090)	18.416	18.416		18.416/18.416			
*L.* × *intermedia* var. ‘Grosso’	*Pseudomonas fluorescens*	3.75	7.51	% (*v*/*v*)	33.38/66.84	–	In vitro liquid/vapour phase screening only; mechanism not experimentally investigated	[53]
	*E. coli*	1.87	1.87		16.64/16.64			
	*Kocuria marina*	1.87	–		16.64/–			
	*Bacillus cereus*	0.94	–		8.37/–			
	*Arthrobacter bohemicus*	0.47	0.47		4.18/4.18			
*L. angustifolia*	*Aeromonas hydrophila*	0.5–1	0.5–2	% (*v*/*v*)	4.45–8.90/4.45–17.80	–	Mechanism not directly evaluated; Pseudomonas aeruginosa exhibited intrinsic resistance (MIC >2%)	[54]
	*Aeromonas caviae*							
	*Aeromonas dhakensis*							
	*Citrobacter freundii*							
	*Proteus mirabilis*							
	*Salmonella enterica*							
	*P. aeruginosa*	>2	–		>17.80/–			
*L. hybrid*	*E. coli*	0–7.1	–	mg/mL	0–7.1/–	–	Focus on microparticle formulation; specific bacterial cell targets not experimentally evaluated	[55]
	*S. aureus*	0–7.1			0–7.1/–			
	*B. cereus*	1.8–3.6			1.8–3.6/–			
*L. angustifolia*	*S. aureus*	1200	–	μg/mL	1.20/–	Enhanced aqueous solubility via HPCD inclusion, facilitating active constituent access to microbial membrane and cytoplasm	In vitro formulation study; in vivo kinetics and cellular pathways not evaluated	[56]
	*E. coli*	2000			2.00/–			
	*S. aureus* (LEO/HPCD)	450			0.45/–			
	*E. coli* (LEO/HPCD)	650			0.65/–			
*L. stoechas* L.	*Aggregatibacter actinomycetemcomitans* (NTCC 9710)	4 (uniform for all 13 strains)	–	μL/mL	3.56/– (uniform for all 13 strains)	Perturbation of cytoplasmic membrane proteins and disintegration of outer membrane lipopolysaccharides	In vitro agar dilution model; lack of in vivo clinical biofilm models	[57]
	*A. actinomycetemcomitans* AHN 24195							
	*Porphyromonas gingivalis* ATCC 33277							
	*P. gingivalis* AHN 24155							
	*P. gingivalis* AHN 24135							
	*Parvimonas micra* ATCC 33270							
	*P. micra* AHC 15107							
	*P. micra* AHC 15154							
	*Tannerella forsythia* AHN 24212							
	*Fusobacterium nucleatum* ATCC 25586							
	*F. nucleatum* AHN 9508							
	*Prevotella intermedia* ATCC 25611							
	*P. intermedia* AHN 8290							
	*Prevotella nigrescens* ATCC 33563							
	*P. nigrescens* AHN 8293							

Notes: – indicates not determined or not reported. To facilitate direct cross-study comparisons, normalized MIC values (mg/mL) were estimated using the standard mean density of lavender essential oil (~0.89 g/mL at 20 °C, per ISO 3515/European Pharmacopoeia standards): 1 μL/mL ≈ 0.89 mg/mL; 1% *v*/*v* ≈ 8.9 mg/mL. Abbreviations: MIC: minimum inhibitory concentration; MBC: minimum bactericidal concentration; ATCC: American Type Culture Collection; DSM: German Collection of Microorganisms and Cell Cultures; HPCD: hydroxypropyl-β-cyclodextrin.

**Table 4:** Antifungal activity of lavender essential oil against different fungal strains.

Lavender Source	MIC	MFC	Original Unit	Normalized MIC/MFC (mg/mL)	Proposed Targets/Modes of Action	Study Limitations	Reference
*L. angustifolia* Mill.	0.69	1.1	% (*v*/*v*)	6.14/9.79	Inhibition of germ tube formation and hyphal elongation, blocking C. albicans dimorphic transition	In vitro cell culture model only; in vivo mucosal/topical efficacy not evaluated	[73]
	1.04	1.8		9.26/16.02			
	0.5	1		4.45/8.90			
*L. vera* DC	0.5–1	–	% (*v*/*v*)	4.45–8.90/–	–	In vitro broth microdilution only; specific underlying antifungal cellular mechanism non-elucidated	[74]
	0.5–1			4.45–8.90/–			
	0.125–1			1.11–8.90/–			
Lavender (Hokkaido, Japan)	400	–	μg/mL	0.40/–	–	In vitro agar dilution and vapour contact assay; specific molecular target interaction not determined	[75]
*L. angustifolia* Mill.	1000	–	μg/mL	1.00/–	–	Limited activity against *P. digitatum* radial growth; lack of mechanistic pathway characterization	[76]

Notes: – indicates not determined or not reported. To facilitate direct cross-study comparisons, normalized MIC values (mg/mL) were estimated using the standard mean density of lavender essential oil (~0.89 g/mL at 20 °C, per ISO 3515/European Pharmacopoeia standards): 1 μL/mL ≈ 0.89 mg/mL; 1% *v*/*v* ≈ 8.9 mg/mL. Abbreviations: MIC: minimum inhibitory concentration; MFC: minimum fungicidal concentration.

**Table 5:** Anti-inflammatory activity of lavender essential oil and its proposed mechanisms.

Lavender Species	Origin	Experimental Model	Inflammatory Outcomes	Cytokine Expression	Oxidative Stress Alleviation	Mechanism of Action	Reference
*L. stoechas* L.	Australian	LPS stimulated THP-1 cells	Cell-to-cell adherence ↓	superoxide anion ↓, IL-1β ↓	–	Suppresses TLR4 expression and NF-κB p65 phosphorylation.	[7]
*L. angustifolia* Mill.	Isfahan	Carrageenan-induced mouse paw edema model	Paw edema ↓	–	–	–	[99]
*L. angustifolia*	Xinjiang	TPA induced inflammation in mouse ear	Ear edema ↓	TNF-α ↓	COX-2 ↓	Suppresses NF-κB activation.	[100]
*L. angustifolia* Mill.	Italy	*Staphylococcus aureus*-infected monocyte-derived macrophages	*–*	IL-6 ↓, IL-1A ↓, TNF-α ↓	HO-1 ↑	Modulates TLR3/TLR8/TLR9 and MAPK-related inflammatory signaling.	[101]
*L. angustifolia*	Brazil	Croton-oil-induced mouse ear edema model and carrageenan- and dextran-induced mouse paw edema model	Ear edema ↓, Paw edema ↓		MPO ↓, NO ↓	–	[102]
*L. angustifolia* Mill.	Brazil	Croton-oil-induced mouse ear edema model and carrageenan-induced pleurisy model with female Wistar rats	Ear edema ↓, Edematogenic response ↓	–	–	–	[103]
*L. × intermedia*	Okanagan	*Citrobacter rodentium* induced colitis in C57BL/6 and C3H/HeOuJ mice	Intestinal tissue damage ↓	TNF-α ↓, IFN-γ ↓, IL-22 ↓	MIP-2α ↓, iNOS ↓	–	[104]
*L. angustifolia*	Osaka	TNF-α-induced cell adhesion molecules in murine brain endothelial bEnd.3 cells and human umbilical vein endothelial cells (HUVECs)	–	VCAM-1 ↓, ICAM-1 ↓	–	Inhibits NF-κB/p65 activation.	[105]

Notes: – indicates not determined or not reported. ↑ indicates increase/upregulation. ↓ indicates decrease/downregulation. Abbreviations: LPS: lipopolysaccharide; NF-κB: nuclear factor kappa B; TNF-α: tumor necrosis factor-alpha; IL: interleukin; TLR: Toll-like receptor; MAPK: mitogen-activated protein kinase; HO-1: heme oxygenase-1; MPO: myeloperoxidase; NO: nitric oxide; iNOS: inducible nitric oxide synthase; COX-2: cyclooxygenase-2; VCAM-1: vascular cell adhesion molecule-1; ICAM-1: intercellular adhesion molecule-1.

**Table 6:** Antioxidant activity of lavender essential oil.

Lavender Source	Source	DPPH/ABTS Assay Values	Original Unit	Normalized IC_50_ (mg mL^−1^)	Reference Antioxidant	Reference
*L. angustifolia* Sevtopolis	Anatolia	91.56/61.23	SC_50_ (μg/mL)	0.0916/0.0612	α-Tocopherol DPPH: 0.0165 mg/mL ABTS: 0.0119 mg/mL	[11]
*L. angustifolia* Yubileina		102.34/71.50		0.1023/0.0715		
*L. angustifolia* Druzhba		97.61/74.08		0.0976/0.0741		
*L. angustifolia* Raya		105.08/67.39		0.1051/0.0674		
*L. angustifolia* Hebar		99.60/65.00		0.0996/0.0650		
*L. angustifolia* Hemus		101.27/67.50		0.1013/0.0675		
*L. × intermedia* Super A		89.81/61.64		0.0898/0.0616		
*L. × intermedia* Grey Hedge		93.20/64.32		0.0932/0.0643		
*L. stoechas* ‘snowman’	Thailand	363.38/397.55	IC_50_ (mg/mL)	363.38/397.55	Trolox DPPH: 0.00034 mg/mL ABTS: 0.00031 mg/mL	[4]
*L. stoechas* ‘white lavender’		361.59/387.46		361.59/387.46		
*L. stoechas* ‘major’		283.31/322.54		283.31/322.54		
*L. stoechas* ‘avonview’		348.35/391.21		348.35/391.21		
*L. stoechas × viridis* ‘St. Brelade’		67.65/89.26		67.65/89.26		
*L. angustifolia* L. (SCE)	Victoria: Australia	63/–	(%)	–	α-Tocopherol 2:6 di-tert-butyl-4-methylphenol	[10]
*L. angustifolia* L. (H)		48/–				
*L. angustifolia* L. (He)		12/–				
*L. angustifolia* L. Ellagance Purple (field-grown)	Szczecin	16.18/38.01	(mg TE g^−1^ DW)	–	Trolox	[121]
*L. angustifolia* L. Ellagance Purple (in vitro)		37.42/82.52				
*L. angustifolia* L. Blue River (field-grown)		23.58/47.49				
*L. angustifolia* L. Blue River (in vitro)		37.20/82.15				
*L. angustifolia* L. Munstead (field-grown)		8.26/28.02				
*L. angustifolia* L. Munstead (in vitro)		35.07/78.53				
*L. × intermedia* ‘Budrovka	Croatian	21.58/–	IC_50_ (mg/mL)	21.58/–	BHT DPPH: 0.01 mg/mL	[122]
*L. angustifolia*		27.67/–		27.67/–		
*L. angustifolia* (Fresh flowers)	Poland	76.22/–	IC_50_ (mg/mL)	76.22/–	Trolox	[123]
*L. angustifolia* (Fresh aerial parts)		77.11/–		77.11/–		
*L. angustifolia* (Dried flowers)		22.11/–		22.11/–		
*L. angustifolia* (Dried aerial parts)		75.42/–		75.42/–		
*L. angustifolia*	Spain	1.35/103.0	TEAC (μmol TE g^−1^ EO)	–	Trolox	[19]
*L. latifolia*		0.42/1.51				
*L. hybrida* cv. Grosso		0.04/1.13				
*L. hybrida* cv. Super		0.216/1.62				

Notes: – indicates not determined or not reported. Normalized IC_50_ (mg/mL) values were calculated for studies reporting half-maximal inhibitory/scavenging concentrations (1000 μg/mL = 1 mg/mL). Metrics initially expressed as percentage inhibition (%), Trolox equivalents per gram dry weight (mg TE g^−1^ DW), or TEAC (μmol TE g^−1^ EO) are designated as “–” in the normalized IC_50_ column to avoid mathematically unsound conversions across fundamentally distinct bioassay dimensions. Abbreviations: DPPH: 2,2-diphenyl-1-picrylhydrazyl; ABTS: 2,2′-azinobis (3-ethylbenzothiazoline-6-sulfonic acid); IC_50_: concentration required for 50% inhibition; SC_50_: concentration required for 50% scavenging activity; TEAC: Trolox equivalent antioxidant capacity; BHT: butylated hydroxytoluene; TE: Trolox equivalent; DW: dry weight; EO: essential oil; SCE: supercritical CO_2_ extraction; H: hydrodistillation; He: hexane extraction.

**Table 7:** Insecticidal effects of lavender essential oil.

Lavender Source	Source	EO Concentration	Normalized Concentration/Dose	Target Organism	Mortality	Reference
*L. officinalis*	–	10% (*v*/*v*) in EtOH	89.00 mg/mL	*Dermatophagoides pteronyssinus* *Pediculus humanus*	87%: at 2 h 22%: at 2 h	[178]
*L. angustifolia*	Iran	8.0% (*w*/*v*)	80.00 mg/mL	*Rhipicephalus annulatus*	100%: 24 h after inoculation	[179]
*L. angustifolia*	–	0.76% (*v*/*v*)	6.76 mg/mL	*Boophilus ocellatus*	50%: at 39.3 min	[180]
Lavender	–	0.12 mg/cm^2^	0.12 mg/cm^2^ (surface dose)	*Dermanyssus gallinae*	>97%: after 48 and 72 h	[181]
*L. stoechas* L.	Ouezzane	0.9 µg/mL	0.0009 mg/mL	*Lutzomyia major*	50%	[177]
		7 µg/mL	0.0070 mg/mL	*Leishmania infantum*		
		>10 µg/mL	>0.0100 mg/mL	*Leishmania tropica*		

Notes: – indicates not determined or not reported. To harmonize concentration expressions across bioassays, normalized concentrations were expressed in mg/mL using the mean relative density of lavender essential oil (~0.89 g/mL at 20 °C, per ISO 3515 standards): 1% *v*/*v* ≈ 8.9 mg/mL; 1% *w*/*v* = 10 mg/mL; 1000 μg/mL = 1 mg/mL. Surface contact doses (mg/cm^2^) are maintained as surface density units due to the distinct exposure assay format. Abbreviations: EO: essential oil; EtOH: ethanol.

## Data Availability

No new data were created or analyzed in this study. Data sharing is not applicable to this article.

## Abbreviations

The following abbreviations are used in this manuscript:

ACD	Allergic contact dermatitis;
CNS	Central nervous system;
COX-2	Cyclooxygenase-2;
EO	Essential oil;
GABA	Gamma-aminobutyric acid;
GC-MS	Gas chromatography–mass spectrometry;
HD	Hydrodistillation;
ICD	Irritant contact dermatitis;
LEO	Lavender essential oil;
LPS	Lipopolysaccharide;
MAPK	Mitogen-activated protein kinase;
MBC	Minimum bactericidal concentration;
MFC	Minimum fungicidal concentration;
MIC	Minimum inhibitory concentration;
NF-κB	Nuclear factor kappa-light-chain-enhancer of activated B cells;
Nrf2	Nuclear factor erythroid 2-related factor 2;
ROS	Reactive oxygen species;
SCE	Supercritical carbon dioxide extraction;
SD	Steam distillation;
SOD	Superoxide dismutase;
TGF-β	Transforming growth factor-beta;
TNF-α	Tumor necrosis factor-alpha;
TRPA1	Transient receptor potential ankyrin 1.
